# Tracking CNS and systemic sources of oxidative stress during the course of chronic neuroinflammation

**DOI:** 10.1007/s00401-015-1497-x

**Published:** 2015-10-31

**Authors:** Agata A. Mossakowski, Julian Pohlan, Daniel Bremer, Randall Lindquist, Jason M. Millward, Markus Bock, Karolin Pollok, Ronja Mothes, Leonard Viohl, Moritz Radbruch, Jenny Gerhard, Judith Bellmann-Strobl, Janina Behrens, Carmen Infante-Duarte, Anja Mähler, Michael Boschmann, Jan Leo Rinnenthal, Martina Füchtemeier, Josephine Herz, Florence C. Pache, Markus Bardua, Josef Priller, Anja E. Hauser, Friedemann Paul, Raluca Niesner, Helena Radbruch

**Affiliations:** German Rheumatism Research Center, Berlin, Germany; Department of Neurology, NeuroCure Clinical Research Center, Clinical and Experimental Multiple Sclerosis Research Center, Charité-Universitätsmedizin Berlin, Berlin, Germany; Institut für Neuropathologie, Charité-Universitätsmedizin Berlin, Berlin, Germany; Institute for Medical Immunology, Charité-Universitätsmedizin Berlin, Berlin, Germany; DZNE-German Center for Neurodegenerative Diseases, Berlin, Germany; Department of Paediatrics I, Neonatology, University Hospital Essen, Essen, 45122 Germany; Experimental and Clinical Research Center, Max Delbrueck Center for Molecular Medicine and Charité–Universitätsmedizin Berlin, Berlin, Germany; Department of Neuropsychiatry and Laboratory of Molecular Psychiatry, Charité-Universitätsmedizin Berlin, Cluster of Excellence NeuroCure and BIH, Berlin, Germany; Intravital Imaging and Immune Dynamics, Charité-Universitätsmedizin Berlin, Berlin, Germany

**Keywords:** Multiple sclerosis, Oxidative stress, Neuronal dysfunction, Fluorescence lifetime microscopy, Intravital imaging, Oxidative stress memory

## Abstract

The functional dynamics and cellular sources of oxidative stress are central to understanding MS pathogenesis but remain elusive, due to the lack of appropriate detection methods. Here we employ NAD(P)H fluorescence lifetime imaging to detect functional NADPH oxidases (NOX enzymes) in vivo to identify inflammatory monocytes, activated microglia, and astrocytes expressing NOX1 as major cellular sources of oxidative stress in the central nervous system of mice affected by experimental autoimmune encephalomyelitis (EAE). This directly affects neuronal function in vivo, indicated by sustained elevated neuronal calcium. The systemic involvement of oxidative stress is mirrored by overactivation of NOX enzymes in peripheral CD11b^+^ cells in later phases of both MS and EAE. This effect is antagonized by systemic intake of the NOX inhibitor and anti-oxidant epigallocatechin-3-gallate. Together, this persistent hyper-activation of oxidative enzymes suggests an “oxidative stress memory” both in the periphery and CNS compartments, in chronic neuroinflammation.

**Electronic supplementary material:**

The online version of this article (doi:10.1007/s00401-015-1497-x) contains supplementary material, which is available to authorized users.

## Introduction

Multiple sclerosis (MS) is a chronic inflammatory neurodegenerative disease characterized by multifocal infiltration of immune cells in the central nervous system (CNS). The first onset of inflammatory symptoms is called clinically isolated syndrome (CIS), and 30–70 % of CIS patients develop definitive MS [34]. In most patients MS has a relapsing–remitting course (RRMS), which is conventionally considered to reflect mainly inflammatory processes. It can, however, subsequently evolve into a secondary progressive disease (SPMS), thought to reflect more of a degenerative process [27, 41], leading to lasting and irreversible neurological disability.

Reactive oxygen species (ROS) generated by invading and resident CNS macrophages have been implicated in mediating demyelination and axonal damage [13, 38, 55, 58, 60]. The term reactive oxygen species (ROS) refers to both free radicals and related molecules. There is evidence pointing to excessive ROS production as a major mechanism causing damage in MS, as oxidized DNA, lipids and proteins were reported in CNS lesions, cerebrospinal fluid and plasma of MS patients [17, 21, 31]. To prevent ROS-caused damage, cells have potent anti-oxidative mechanisms. However, defects in anti-oxidant mechanisms and excessive ROS production that overwhelms the endogenous anti-oxidant capacity lead to oxidative stress. The most abundant source of ROS leading to oxidative stress is the respiratory burst, which is mediated by the nicotinamide adenine dinucleotide phosphate (NADPH) oxidases [NOX1, NOX2 (phox), NOX3, NOX4, DUOX1, DUOX2] [5]. The NOX enzymes catalyze the oxidation of molecular oxygen to its highly reactive anion O_2_^–^. Recently, significant up-regulation of NOX2 as well as histological co-localization of cytosolic and membrane-bound NOX2 subunits have been described in MS, at lesion sites [13, 59]. Additionally, subunits of NOX2 are up-regulated in active lesions in experimental autoimmune encephalomyelitis (EAE), an animal model of MS [45]. Furthermore, other members of the NADPH oxidase family, including NOX1 and NOX4 have the capacity to cause oxidative stress in the CNS [11, 30, 36, 43, 49]. NOX1 subunits have been described in active lesions in MS tissue in microglia/macrophages as well as in astrocytes [13], and inhibition of NOX enzymes [9] and knockout of isotype-specific as well as isotype-common NOX subunits (gp91 [29], p47 [57]) decreased EAE severity and incidence. This suggests that NOX enzymes in general are major contributors to oxidative stress by catalyzing the production of ROS, thus leading to CNS tissue damage. However, up to now, neither the catalytic activity of NOX enzymes leading to oxidative stress nor their cellular sources could be tracked, in vivo in the CNS and in the periphery.

Given the increasingly recognized importance of oxidative stress in MS pathogenesis, anti-oxidant drugs are emerging as new therapeutic approaches [15]. One potential candidate is epigallocatechin-3-gallate (EGCG), a polyphenol present in green tea. We and others have shown that EGCG ameliorates EAE [2, 19, 22, 33, 61], and clinical trials testing the benefits of EGCG in MS patients are ongoing (NCT00525668, NCT00799890 and NCT01417312). There is in vitro data pointing to the inhibition of ROS production through EGCG [63]. It is known that EGCG competes with NADPH but not with NADH in binding to NADH-/NADPH-dependent enzymes [46]. However, the therapeutic effects of EGCG in vivo have not yet been analyzed on a cellular level.

Developed two decades ago, fluorescence lifetime imaging (FLIM) has proven to be a reliable, quantitative tool to probe cellular parameters [1, 12, 24, 25, 35, 53]. In particular, the possibility to quantify the endogenous fluorescence of NAD(P)H or of flavoproteins has made FLIM an especially versatile probe of metabolic function [26, 51, 52], as this marker-free approach allows for the acquisition of unaltered, real-time data. Despite its power as a quantitative technique, FLIM requires a high fluorescence signal and thus could not be applied to deep-tissue imaging until recently [4]—a limitation which is particularly relevant to intravital imaging of protected, hardly accessible organs like the CNS [44]. In our previous work, we demonstrated that, in the context of oxidative stress, the increase of fluorescence lifetime of NAD(P)H is specific for NADPH (and not NADH) binding to members of the NOX family, both in murine polymorphonuclear cells [37] and in plant cells (*Nicotiana tabacum*) (unpublished data). These results are supported by recent studies showing the same increase in NAD(P)H fluorescence lifetime, in a more general context [6]. When using NAD(P)H-FLIM, all NADH- and NADPH-dependent enzymes which participate in biochemical reactions within the cell are simultaneously detected (up to several hundred enzymes). Thus, the activation of NOX enzymes can be unequivocally detected only at sites where these enzymes preferentially bind the present coenzymes. The catalytic activity corresponding to such a situation implies an excessive ROS production, and thus oxidative stress, rather than a low, e.g., immunomodulatory, ROS production.

In the present study, we observe a spatio-temporal correlation between the catalysts of oxidative stress (activated NOX enzymes) and neuronal damage in the CNS of mice affected by EAE. Furthermore, we validate this technique in the CNS by identifying macrophages and activated microglia as major cellular sources of activated NOX enzymes, and thus of oxidative stress, in the brain stem of mice with EAE. Using this novel technique, we show for the first time that astrocytes are also major contributors in generating oxidative stress in the CNS during chronic EAE. Additionally, we offer insight into the systemic dimensions of both EAE and MS by quantifying the activity of NADPH oxidases in peripheral monocytes, from the first presentation of symptoms to the chronic phase of the disease. This marker-free approach allows the direct monitoring of the therapeutic mechanism of the anti-oxidative drug EGCG in humans at a cellular and even sub-cellular level.

## Methods and materials

### Two-photon laser-scanning microscopy (TPLSM)

FLIM experiments were performed using a specialized two-photon laser-scanning microscope based on a commercial scan head (TriMScope, LaVision BioTec, Bielefeld, Germany) [20, 44]. The detection of the fluorescence signals was accomplished either with photomultiplier tubes in the ranges 460 ± 30, 525 ± 25, 593 ± 20 nm or with a 16-channel parallelized TCSPC detector (FLIM-X_16_, LaVision BioTec, Bielefeld, Germany) in the range 460 ± 30 nm. The excitation of NADH and NADPH was performed at 760 nm (detection at 460 ± 30 nm), of Cerulean (detection at 460 ± 30 nm), sulforhodamine 101 (detection at 593 ± 20 nm) and EGFP (detection at 525 ± 25 nm) at 850 nm and of tdRFP either at 930 nm or at 1110 nm (detection at 593 ± 20 nm). Citrine was detected at 525 ± 25 and 593 ± 20 nm.

For both intensity and fluorescence lifetime imaging we used an average maximum laser power of 8 mW to avoid photodamage. The experimental parameters for FLIM were 160 ps histogram bin (for NAD(P)H-FLIM) and 80 ps histogram bin (for FRET-FLIM) and maximum acquisition time for a 512 × 512 image was 5 s to record a fluorescence decay stack (Suppl. Fig. 3). The time window in which the fluorescence decays were acquired was set to 9 ns.

### Data analysis

FLIM data analysis was performed using self-written software based on Levenberg–Marquardt algorithms for non-linear fitting as well as the phasor approach (Suppl. Fig. 6).

Statistical analysis and graphical presentation was carried out with GraphPad Prism 4 (Graphpad Software, USA) and OriginPro (OriginLab, USA). Results are shown as mean values from analyzed data in one mouse/human, in addition the mean ± SD summarize collective data from performed experiments.

### Mice

All mice used were on a C57/Bl6 background. The *CerTN L15 x LysM tdRFP* mouse expresses a FRET-based calcium biosensor consisting of Cerulean (donor) and Citrine (acceptor) bound to troponin C, a calcium-sensitive protein present in certain subsets of neurons [18]. Mice carrying the CerTN L15 reporter were crossed to the LysM tdRFP mice, in which tdRFP [32] is expressed in LysM^+^ cells [10], yielding progeny with both reporters. The *CX*_*3*_*CR*_*1*_^+*/*−^*EGFP* mouse [23] was used to detect microglia, whereas the *CD4*^+^*YFP* mouse strain was used to detect CD4^+^ T cells [28, 50]. EAE induction protocol and EAE course of these mice can be found in Table 2, Supplemental Material.

### Preparation of the brain stem window for intravital imaging

The preparation of the imaging field was similar to our previous description [47, 48]. For a detailed description, see Supplemental Material. In each imaged animal the whole area that was accessible by intravital imaging was scanned and all visible lesions of each animal were analyzed as well as at least one “normal appearing” imaging field. Animal experiments were approved by the appropriate state committees for animal welfare (G0198/11 and G0181/10, LAGeSo—Landesamt für Gesundheit und Soziales Berlin) and were performed in accordance with current guidelines and regulations.

### Human samples

Venous blood samples were obtained with informed consent after the nature and possible consequences of the studies were explained. We included healthy controls, patients with untreated clinically isolated syndrome (CIS), patients with relapsing remitting MS (RRMS-GA) in a randomized double-blind clinical trial medicated with glatiramer acetate receiving additional 600 mg of EGCG or placebo, patients with relapse and remitting MS not treated with any immunomodulatory drugs (RRMS), and patients with a secondary progressive disease course (SPMS); further detailed information can be found in Table I, Supplemental Material. Peripheral blood mononuclear cells (PBMCs) were isolated using standard protocols and CD11b^+^-enriched by MACS selection (Miltenyi Biotec, Bergisch Gladbach, Germany) according to the manufacturer’s protocol. The purity was assessed by flow cytometry. The MACS-enriched CD11b^+^ monocytes were injected into specifically fabricated chambers and incubated in phenol-free RPMI at 37° C for one hour prior to imaging.

Further experimental details are available in Supplemental Methods.

## Results

### Activation of NOX enzymes measured by intravital NAD(P)H fluorescence lifetime imaging pinpoints the origin of oxidative stress and correlates with immune infiltration and neuronal dysfunction in EAE

**Fig. 1:**
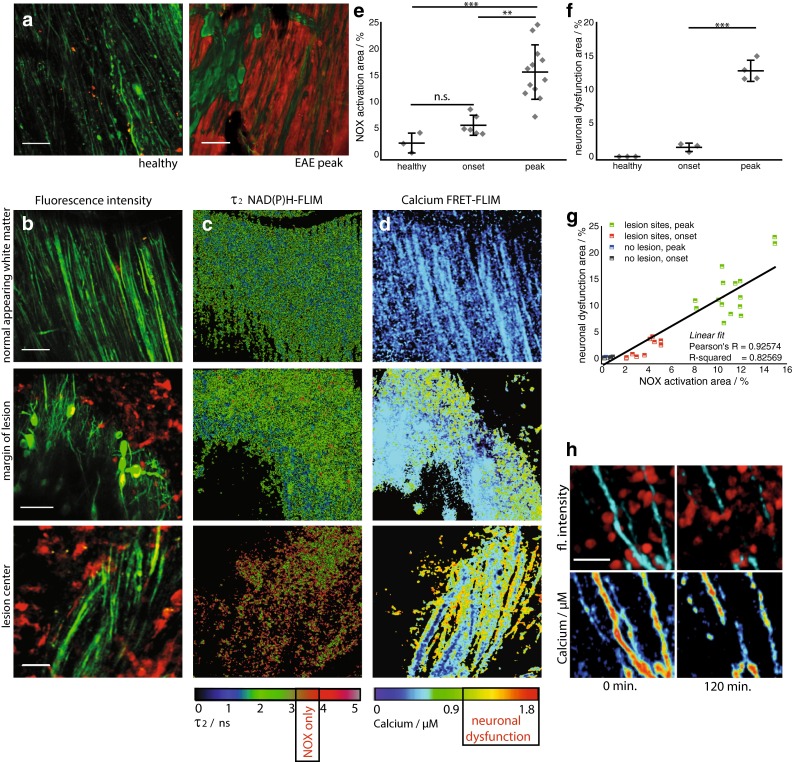
Activation of NADPH oxidases in the CNS pinpoints oxidative stress. Oxidative stress is dependent on disease severity and correlates with cellular inflammation and neuronal dysfunction. ROS concentration in the brain stem of a healthy mouse compared to a mouse affected by EAE at peak of disease (score 2.0) (**a**). Amplex Red^®^ was used as ROS indicator. *λ*_exc_ = 910 nm, *λ*_em_ = 525 ± 25 nm (Thy1-Citrine in neurons depicted in *green*), *λ*_em_ = 593 ± 20 nm (Amplex Red^®^), *scale bar* 50 µm. **b** Correlated 300 × 300 µm^2^ (517 × 517 pixel) fluorescence intensity images [Thy1 = *green* (neurons), LysM = *red* (macrophages)], **c** enzyme-bound NAD(P)H-FLIM maps (τ_2_-maps) and **d** neuronal Ca^2+^ maps as recorded by FRET-FLIM in three different regions of the brain stem of a *CerTN L15 x LysM tdRFP* mouse with EAE, at the peak of the disease, score = 2.5. *First row* normal appearing white matter; *second row* margin of lesion; *third row* lesion center. The activation of the NADPH oxidases (NOX) assessed by NAD(P)H-FLIM (**c**) correlates with LysM^+^ tdRFP cell infiltrate in the CNS (**b**) and with elevated neuronal calcium (**d**), indicating neuronal dysfunction. *Scale bars* in **b**, **c** and **d** = 30 µm. The τ_2_-maps show the false color-encoded fluorescence lifetime τ of enzyme-bound NAD(P)H at each recorded pixel of the image. NAD(P)H bound to metabolic enzymes are depicted in blue and green (τ between 1 and 3 ns) whereas, under oxidative stress, NADPH bound to activated NOX appears in red (τ between 3.3 and 3.9 ns, “NOX only” gate). **e** Quantification of the NOX activation area within lesions defined by LysM^+^ cell infiltration, i.e., ratio of the area of NOX only gate to the total tissue area, 4.98 ± 1.53 % at the onset of clinical signs (day 1–2 after appearance of first clinical signs, EAE scores 0.5–1.0) and 14.88 ± 5.81 % of the tissue area at peak of disease (day 3–5 after onset of clinical symptoms, EAE scores 1.5–2.5) as compared to 2.19 ± 0.94 % in healthy controls. Six independent EAE experiments (EAE onset *n* = 6, EAE peak *n* = 12 healthy controls *n* = 3), see Table 2. **f** Quantification of elevated neuronal calcium area is assessed by FRET-FLIM relative to the total neuronal area. Data display means of imaging fields of individual animals from two independent EAE experiments (healthy controls *n* = 3, EAE onset *n* = 3, EAE peak *n* = 4). The increased NOX activity correlates with an increased area of elevated neuronal calcium (“Ca^2+^ > 1 µM” gate) at the time of EAE onset (1.32 ± 0.78 %) and at peak disease (12.80 ± 2.41 %), as compared to healthy controls (0 %, *n* = 3, mean calcium concentration 170 nM, maximum calcium concentration 450 nM). **g** Correlation between NOX enzymes activation area and increased neuronal calcium area at lesion sites and non-infiltrated sites in the brain stem of *CerTN L15 x LysM tdRFP* mice affected by EAE. **h** Pathologically increased neuronal calcium level (>1 µM calcium) correlates with immune cell infiltrate and, after 2 h, with axonal break-down as shown by intravital images in the brain stem of a *CerTN L15* mouse affected by EAE (peak)—*scale bar* 10 µm. All images are acquired in the region 30–150 µm depth within the brain stem (300 × 300 × 50 µm^3^, z-step = 2 µm). For the statistic evaluation in **e** and **f** we applied ANOVA tests (*p < 0.05, **p < 0.01, ***p < 0.001)

To detect and quantify NOX enzymes activation in vivo, we induced EAE in mice and imaged their brainstem intravitally. Consistent with previous reports [38], we detected elevated ROS concentrations (~200 µM) in the brain stem of mice affected by EAE at the peak of disease, compared to disease-free control animals (Fig. 1a). ROS were detected by intravital imaging of murine brainstems which had been previously superfused with a 100 µM solution of Amplex Red^®^. The Amplex Red^®^ calibration was performed using a H_2_O_2_ concentration series (Suppl. Fig. 2).

As the main catalysts of O_2_^−^ production (precursor of ROS) the membrane-bound NOX enzymes (NOX1,2,3,4 and DUOX1,2) directly contribute to oxidative stress. By employing intravital NAD(P)H fluorescence lifetime imaging (FLIM) we detected the activation of NOX enzymes in the brainstem of EAE mice (Table 2). From the fluorescence intensity decay of NAD(P)H, we evaluated the fluorescence lifetime of enzyme-bound NAD(P)H at each pixel of the image in different layers of the brainstem between 30 and 150 µm depth, as described in Supplemental Material. We expected a mean fluorescence lifetime between 1.1 and 2.7 ns for NAD(P)H bound to metabolic enzymes responsible for basic cellular functions (Suppl. Fig. 4) and a fluorescence lifetime of approximately 3.65 ns for NADPH bound to NOX enzymes as calculated with two independent evaluation methods [37] (Supplemental Material, Suppl. Figs. 4 and 6). Free NAD(P)H has a fluorescence lifetime of approx. 400 ps and is easily distinguished from enzyme-bound NAD(P)H.

The specific NAD(P)H fluorescence lifetime (“NOX only” gate set at 3.3–3.9 ns) corresponding to activated NOX enzymes was detectable in EAE mice mainly within those brainstem regions that included immune cell infiltrations (LysM^+^tdRFP or CD4^+^YFP cells, Movie 1); that is, NOX enzymes overactivation was detected in acute lesions, but could not be detected in “normal appearing” areas in the same animal (peak disease, score 2.5, Fig. 1b, c).

The area of NOX-specific activation in acute lesions in the brainstem was significantly enlarged, both at the onset of clinical signs, with a mean of 5 ± 1.5 % in the field of view (300 × 300 µm^2^), and at the peak of disease, with a mean area of 14.9 ± 5.8 %, as compared to healthy animals, with a mean area of 2.2 ± 0.9 % (Fig. 1e).

By performing intravital FRET-FLIM in the brain stem of *CerTNL15xLysMtdRFP* mice with EAE, we were able to confirm and extend our previous results indicating neuronal dysfunction during the disease (Fig. 1d, f). We showed previously that elevated Ca^2+^ concentrations over 1 µM in these mice over a period of 1 h reliably indicate neuronal dysfunction [48]. Here, we confirm this finding by showing that neuronal and axonal calcium is increased in areas adjacent to immune infiltrates. Additionally, after 2 h of intravital monitoring, the sites of increased axonal calcium showed axonal disintegration (Fig. 1h). Both the TN L15 construct decay time and the acquisition time of the FRET-FLIM technique are in the range of several 100 ms, allowing for recording time-averaged calcium concentrations relevant for the pathologic dysfunction of neurons. They are, however, both too slow to monitor neuronal calcium transients.

By simultaneously performing NAD(P)H-FLIM and FRET-FLIM in the brainstems of mice with EAE, we found that the increased NOX activation correlated with an extended area of elevated neuronal calcium (“Ca^2+^ >1 µM” gate) at EAE onset (1.3 ± 0.8 %) and at peak disease (12.8 ± 2.4 %), as compared to control mice (0 %) (Fig. 1g). The high spatial correlation between areas of NOX enzymes activation and increased neuronal calcium indicating early neuronal dysfunction (Pearson’s *R* coefficient over 0.9) in EAE is depicted in Fig. 1g. Interestingly, at lesion sites, both the overactivation of NOX enzymes and the neuronal dysfunction were generally lower at onset than at disease peak. The areas of NOX enzymes activation and increased calcium values were determined as mean values of 3–12 acute lesions per mouse. Each data point in Fig. 1e, f represents the average value for each individual mouse.

### Cellular origins of oxidative stress in EAE

We verified the capacity of intravital NAD(P)H-FLIM to identify the cellular origins of oxidative stress directly in the CNS by confirming the contributions of macrophages and activated microglia, which are well-known contributors to the activation of NOX enzymes during EAE. Subsequently, we used the unique versatility of this technique to identify novel cellular sources of oxidative stress by performing endogenous NAD(P)H-FLIM in the CNS of EAE mice with a variety of fluorescently labeled cell subsets. Here, we evaluated the relative contributions of each cell type (LysM^+^tdRFP, CerTNL15, CD4^+^YFP, CX_3_CR_1_^+/−^EGFP or sulforhodamine101 labeled cells) to the total area of NOX enzymes activity.

### Characterization of cellular markers

**Fig. 2:**
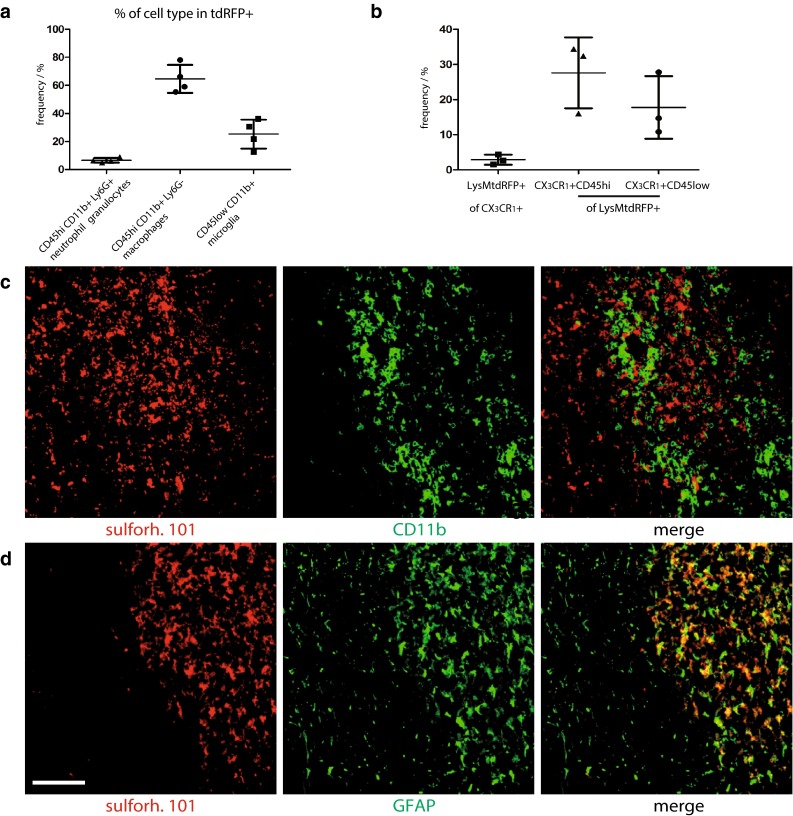
Characterization of active lesions as well as of LysMtdRFP^+^ and sulforhodamine 101 labeled cells during EAE. **a** FACS analysis data in the CNS of LysM^+^tdRFP mice at peak EAE, characterizing the tdRFP^+^ cell population (*n* = 4). **b** FACS analysis data in the CNS of LysM^+^tdRFP mice (*n* = 3) at peak EAE showing the overlap of LysMtdRFP and CX_3_CR_1_-antibody labeled cells. **c** Immunofluorescence images within the brain stem of a C57Bl6 mouse affected by EAE, at peak of the disease, showing no co-localisation between sulforhodamine 101 (*red*) and CD11b^+^ cells (*green*) at infiltration sites. **d** Immunofluorescence images within the brain stem of the same mouse, showing colocalization between sulforhodamine 101 (*red*) and GFAP^+^ cells (*green*) at reactive gliosis sites. *Scale bar* 200 µm

The reliability of the chosen cellular markers used in the intravital experiments was verified by flow cytometry or histology in mice affected by EAE. While in healthy LysM^+^tdRFP animals only rare tdRFP^+^ cells can be found in the CNS, during EAE (*n* = 4) the tdRFP^+^ compartment increased markedly at lesion sites. Of the tdRFP^+^ cells, 65 % were CD45^high^CD11b^+^Ly6G^−^ (macrophages), 25 % CD45^low^CD11b^+^ (microglia) and 7 % CD45^high^CD11b^+^Ly6G^+^ (neutrophil granulocytes) (Fig. 2a). The overlap of LysM^+^tdRFP cells with CX_3_CR_1_^+^ cells was quantified by flow cytometry using an anti-CX_3_CR_1_ antibody. 17.8 ± 8.9 % of all LysMtdRFP^+^ cells were CX_3_CR_1_^+^CD45^low^ (microglia) and 27.6 ± 10.1 % were CX_3_CR_1_^+^CD45^high^ (macrophages). This represented only a minority of the total CX_3_CR_1_^+^ cells as displayed in the 2.8 ± 1.4 % of cells that co-expressed LysMtdRFP (*n* = 3) (Fig. 2b).

**Fig. 3:**
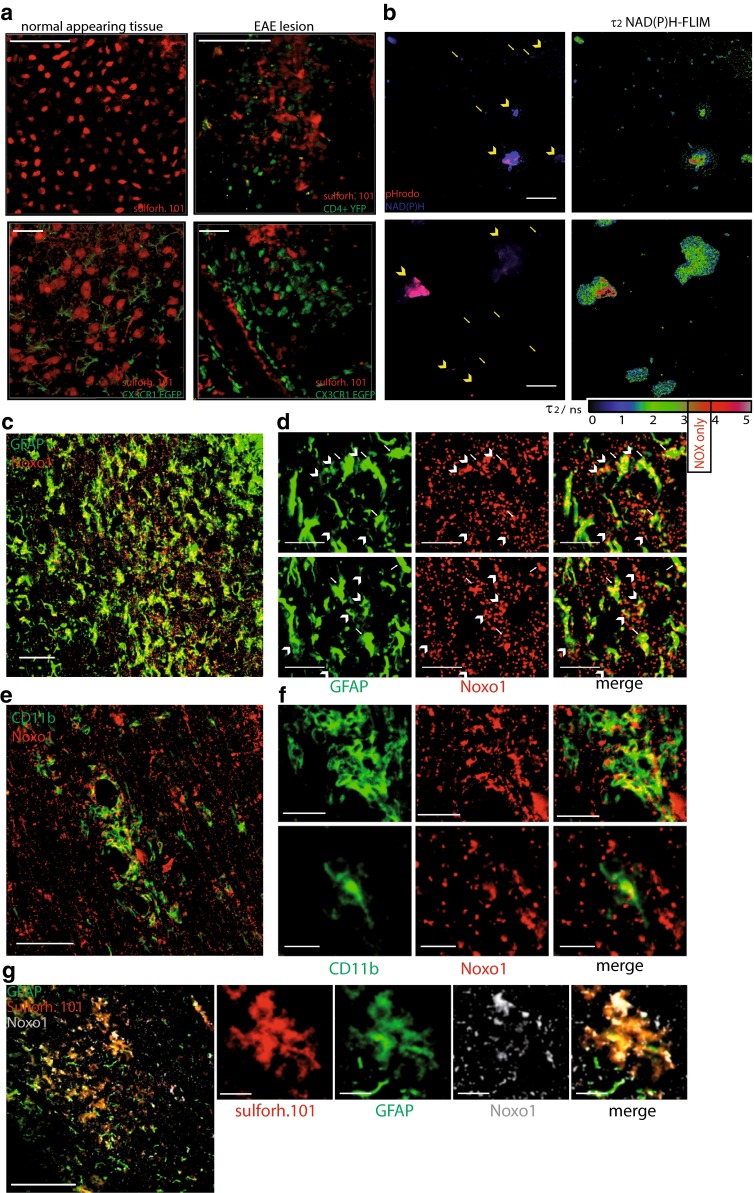
Astrocytes as phagocytes and their potential role in EAE. **a** 3D fluorescence intensity images were acquired in the brain stem of CD4^+^ YFP mice: healthy controls (*upper*, *left panel*) and at a lesion site, in EAE (*upper*, *right panel*) [CD4^+^ cells (YFP) = *green*, astrocytes (sulforhodamine 101) = *red*]. *Scale bar* 100 µm. Similarly, 3D fluorescence intensity images were acquired in the brain stem of CX_3_CR_1_^+/−^ EGFP mice: healthy controls (*lower*, *left panel*) and at the lesion site in EAE (*lower*, *right panel*) [microglia/macrophages (EGFP) = *green*, astrocytes (sulforhodamine 101) = *red*]. *Scale bar* 30 µm. **b** NAD(P)H fluorescence images (*blue*) of mixed astrocytes (*arrow head*) and microglia (*arrows*) cell cultures phagocytosing *Staphylococcus aureus* beads (pHrodo, *red* after uptake) are shown on the *right side*. Corresponding NAD(P)H-FLIM maps (τ_2_-maps) shown on the *left side* reveal the correlation between phagocytosis of pHrodo beads and the typical increase of fluorescence lifetime in astrocytes indicating the activation of NOX enzymes. **c** Immunofluorescence overview image within the brain stem of a mouse affected by EAE indicating the distribution of Noxo1 (subunit of NOX1, *red*) and GFAP signal (*green*). *Scale bar* 100 µm. **d** Zoom ins of **c** demonstrating that Noxo1 is found both in GFAP^high^ cells (indicated by *white arrows*) and GFAP^low^ cells (indicated by *white arrow heads*) in the CNS of mice affected by EAE. *Scale bar* 30 µm. **e** Immunofluorescence overview image within the brain stem of a mouse affected by EAE indicating the distribution of Noxo1 (subunit of NOX1, *red*) and CD11b signal (*green*). *Scale bar* 150 µm. **f** Zoom ins of **e** demonstrating that Noxo1 is found in CD11b^+^ cells (with both phagocytic and microglial morphology). *Scale bar* 40 µm (*upper panels*), *scale bar* 20 µm (*lower panels*). **g** Immunofluorescence overview image within the brain stem of a mouse affected by EAE indicating the distribution of Noxo1 (subunit of NOX1, *gray*), GFAP signal (*green*) and sulforh101 signal (*red*). *Scale bar* 200 µm. **h** Zoom in of **g** demonstrating that Noxo1 is found in GFAP^low^, sulforh101-labeled cells. *Scale bar* 30 µm

In disease-free control animals, sulforhodamine 101 (sulforh101) specifically labels astrocytes in the cortex [39, 40]. To characterize the cells labeled with sulforh101 during EAE in the brainstem we analyzed by immunofluorescence intracellular sulforh101 (following i.v. injection) as well as GFAP and CD11b expression. Minimal co-localization (6.1 ± 1.4 %) of sulforh101 labeling with CD11b staining could be observed at EAE lesion sites in the brainstem (Fig. 2c). We detected sulforh101 co-localization mainly with GFAP^low^ cells [16] but not with GFAP^high^ cells (Fig. 2d). 80 ± 6 % of sulforh101-labeled cells were GFAP^+^, whereas this cell population represents only half of all GFAP-expressing cells (Fig. 2d). Intravital imaging data recorded in the brainstem of CX_3_CR_1_^+/−^EGFP mice additionally labeled with sulforh101 showed negligible overlap between the EGFP and sulforh101 labeling, typically 2 ± 1.5 %, both in healthy controls and in mice affected by EAE (Fig. 3a). The lesions investigated by intravital imaging in the brainstem showed typical EAE characteristics, which we confirmed by histology (Suppl. Fig. 1).

### Phagocytic capacity of astrocytes and their potential role in EAE

We focused on astrocytes as potential contributors to oxidative stress during neuroinflammation as they are known to have phagocytic capacity. Morphological analysis of our intravital experiments revealed a disruption of the fine process-rich astrocytic network typically found in healthy control animals (Fig. 3a), similar to that which could be seen on histological staining (Fig. 2). The disruption of the astrocytic network was associated with changes towards a reactive phenotype and glial scar formation at lesion sites, with dense accumulations of astrocytic end feet and processes lining up along the vessels and around inflammatory cells (CX_3_CR_1_^+^) in EAE (Figs. 2d, 3a).

Furthermore, NAD(P)H-FLIM analysis in astrocytes using beads for phagocytosis coated with *Staphylococcus aureus* fragments (pHrodo) in mixed cell cultures with microglia showed increased NAD(P)H fluorescence lifetime typical of NOX enzymes activity. We could correlate increased NOX enzymes activation with the localization of phagocytosed pHrodo beads (red) (Fig. 3b).

As the expression of p22 NOX subunit in astrocytes during EAE is only sparse [45], we investigated Noxo1 expression by immunofluorescence to address the question which NOX enzyme could be responsible for the observed NAD(P)H lifetime prolongation. NOX1 function is less dependent on p22 coexpression compared to other isoforms [54]. We could detect in GFAP^high^ cells and GFAP^low^ cells Noxo1 expression (Fig. 3c, d) including GFAP^low^ and sulforh101-co-labeled cells (Fig. 3g) in EAE lesions. Furthermore, Noxo1 was also found in CD11b^+^ cells (with both phagocytic and microglial morphology) (Fig. 3e).

Altogether, these data suggest that astrocytes generate oxidative stress within the CNS during the course of chronic neuroinflammation.

### Astrocytes are major cellular contributors to oxidative stress in EAE

**Fig. 4:**
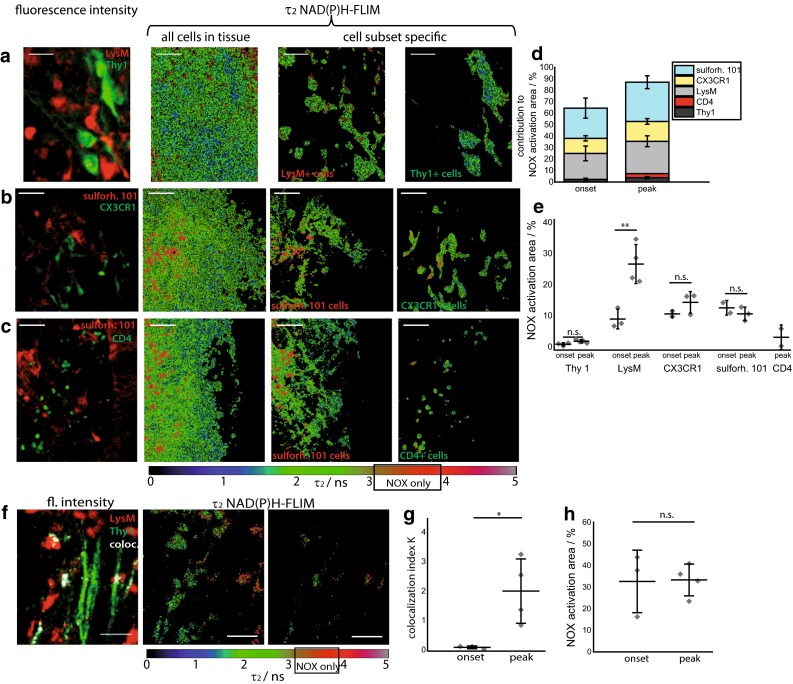
Cellular origin of NADPH oxidases activity in the CNS of EAE mice. **a** The fluorescence intensity image and the τ_2_ NAD(P)H-FLIM map of whole tissue area was used to analyze the cellular origin of NOX activation signal in the CNS. Therefore, we performed an overlay of both images and analyzed the NAD(P)H-FLIM signal at the areas of the respective cell types. Exemplarily LysM^+^ cells as well as Thy1^+^ cells (neurons) in **(a)**, CX_3_CR_1_^+/−^ EGFP—*green* or sulforhodamine 101—*red* (astrocytes) in **b** and CD4^+^ YFP—*green* (T cells) or sulforhodamine 101—*red* (astrocytes) in **c** are shown in the brain stem of mice affected by EAE. All τ_2_ NAD(P)H-FLIM images depict the normalized area of NOX activation in relation to the total cellular area in the respective cell subsets. *Scale bar* 30 µm (**a**) and 50 µm (**b**, **c**). Quantification of the contribution of LysM, Thy1, CX_3_XR_1_, CD4^+^ and sulforhodamine 101-labeled cell subsets, respectively, to the total area of NOX enzymes activation within a lesion. LysM^+^ phagocytes: 22.7 % at onset and 28.2 % at the peak of EAE, CX_3_CR_1_^+^ cells: 13.1 % at onset and 17.4 % at the peak of EAE, astrocytes (sulforhodamine 101): is 26.2 and 37.0 %, CD4^+^ cells: 3.6 % at peak disease, neurons (Thy1^+^): 2.3 % at onset and 3.8 % at peak (**d**). To analyze the proportion of activated cells in one specific cell subset, we quantified the NOX enzymes activation area relative to the total area of the respective cell type at onset and peak of disease (**e**). The area of NOX activation within neurons (Thy1^+^ cells in the *LysM* ^+^ *tdRFP x CerTN L15* mice): onset 0.8 ± 0.5 % and peak 1.8 ± 0.4 %, LysM^+^ cells onset 7.9 ± 2.3 %, and peak 26.4 ± 6.6 %, CX_3_CR_1_^+^ cells: onset 10.6 ± 0.6 % and peak 14.4 ± 1.7 %, astrocytes (sulforhodamine 101): onset 11.7 ± 0.6 % and peak 9.8 ± 1.6 %, CD4^+^ cells: peak 3.1 ± 2.0 %. Data from 2 EAE experiments in *LysM* ^+^ *tdRFP x CerTN L15* mice, *n* = 3 onset, peak *n* = 4; 2 EAE experiments in *CX*_*3*_ *XR*_*1*_^+*/*−^ *EGFP* mice labeled with sulforhodamine 101, *n* = 2 onset and *n* = 3 peak; 1 EAE experiment in *CD4* ^+^ *YFP* mice labeled with sulforhodamine 101, peak *n* = 2 (**e**). *Left* fluorescence intensity image of brain stem lesion in EAE (Thy1 = *green*, LysM = *red*, colocalisation = *white*). τ_2_ (enzyme-bound) NAD(P)H-FLIM image of LysM^+^tdRFP cells in tissue (*middle*) and at the Thy1/LysM colocalisation area (*right*) depicts the normalized area of NOX enzymes activation in relation to total LysM^+^ cellular area as well as the activation within the colocalisation area. *Scale bar* 30 µm (**f**). The normalized colocalisation index indicates the interaction level between LysM^+^ and Thy1 cells, respectively, in onset and in peak of the disease, in the same experiments (**g**). Quantification of the mean NOX activation area of individual mice relative to the total area of LysM/Thy1 colocalisation at the onset (*n* = 3) and peak (*n* = 4) of disease, two independent EAE experiments (**h**). All images are acquired in 30–150 µm depth within the brain stem (z-step = 2 µm). For statistic evaluation in **d** we applied the ANOVA test, in **g** and **h** Mann–Whitney *U* tests (*p < 0.05, **p < 0.01, ***p < 0.001)

We quantified the contribution of specific cell types to the total area of NOX enzymes activity in the CNS using the cellular markers described above. In line with previous studies, we found that the mean contribution of LysM^+^ cells amounted to 22.7 ± 6.4 % at onset, and 28.2 ± 4.7 % at the peak of EAE. Additionally, the mean contribution of CX_3_CR_1_^+^cells amounted to 13.1 ± 2.3 % at onset, and 17.4 ± 2.4 % at the peak of EAE. Macrophages and microglia (either LysM^+^ and/or CX_3_CR_1_^+^) together constitute the main source of oxidative stress at onset and peak of the disease (Fig. 4d). This finding confirms the reliability of our method to evaluate oxidative stress by detecting the activation of NOX enzymes.

Using this novel approach, we found that although microglia and macrophages are the main sources of NOX enzymes activity, a significant fraction could not be attributed to those cell types. Surprisingly, we found that astrocytes (sulforhodamine 101-labeled cells [3]) accounted for 26.4 ± 4.4 and 34.2 ± 5.6 % of total area of NOX enzymes overactivation at onset and peak of EAE, respectively (Fig. 4d). In this model, the contribution of astrocytes to oxidative stress within the CNS is comparable to that of the labeled macrophages and microglia together, thus identifying astrocytes also as central contributors to oxidative stress in the acute phases of EAE.

The mean contribution of CD4^+^cells to NOX enzymes activity was negligible, amounting to 3.6 % at peak disease, comparable to the mean contribution of neurons with 2.3 % at onset and 3.8 % at peak of EAE (Fig. 4d).

We further analyzed the fraction of activation within each cell subset by calculating the percentage of the specific cell area that shows NOX enzymes activation, as well as averaged values per mouse displayed in Fig. 4e. The area of NOX enzymes activation within neurons (Thy1^+^ cells in *LysM*^+^*tdRFPxCerTNL15* mice) was very small, both at clinical onset and at peak of disease with less than 2 %. Conversely, in LysM^+^tdRFP cells the area of NOX enzymes activation was generally higher with 8 % at clinical onset and rising significantly to 27 % at peak disease (Fig. 4e). The area of NOX enzymes activation in CX_3_CR_1_^+/−^EGFP cells was 11 and 14 %, respectively. We obtained similar data in astrocytes with 12 % at onset and 10 % in peak. In CD4^+^YFP cells, the area of NOX enzymes activation was low at peak of disease, amounting to only 3 %.

In our experimental setup, not all cells of the specific cell subtype are fluorescently labeled also due to the use of heterozygous fluorescent mouse strains, but we could address the majority of cells with our labeling strategy. The remaining percentage of NOX enzyme activation area that was not associated with labeled cells in this model (12.85 % at peak, 35.46 % in onset of EAE) is probably due to unlabeled macrophages, microglia and/or astrocytes. The contribution of other immune or CNS-specific cell types, such as oligodendrocytes or immune cells with phagocytic capacity, to the generation of oxidative stress cannot be completely excluded but is expected to be low.

### Soluble factors drive activation of NADPH oxidases in EAE

Regarding the co-localization of neuronal structures (Thy1^+^ cells) and LysM^+^ cells in *CerTNL15xLysMtdRFP* mice, we found that only a 28 to 30 % of the co-localized area was associated with increased NOX enzymes activation in EAE (Fig. 4f, h). The normalized co-localization index, calculated as we previously described [48] indicated significantly more extensive interaction between neurons and macrophages at peak disease than at the time of onset (Fig. 4g), independently of the number of neurons or macrophages at the imaged site. Thus, while the interactions between neurons and LysM^+^ cells were more extensive with the progression of the disease, NOX enzymes activation was not specifically induced by neurons or by their direct interaction with LysM^+^ cells. On the other hand, we showed that upon applying glutamate (300 µM) locally to the brainstem of healthy mice, the NOX enzymes activation area increased from values typical for control animals (0.7 ± 0.2 %) to values similar as those reached in peak of EAE (11.9 ± 3.1 %) (Suppl. Fig. 5), indicating that glutamate-induced excitotoxicity can lead to similar levels of NOX enzymes activation as are present in acute EAE lesions. These data support the interpretation that the cause of NOX enzymes activity in macrophages, activated microglia and astrocytes within the CNS in EAE is not necessarily triggered by direct contact to CNS components like axons, but is rather associated with soluble factors, such as glutamate or inflammatory mediators.

### NOX is overactivated in peripheral CD11b^+^ monocytes in both MS and EAE and can be ameliorated by systemic administration of EGCG

**Fig. 5:**
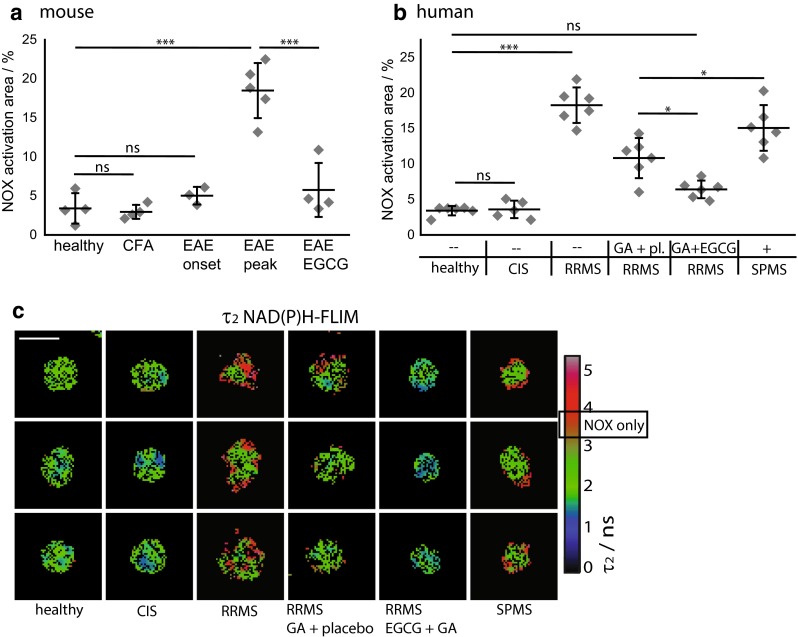
NOX activation in murine and human peripheral CD11b^+^ cells during chronic neuroinflammation as compared to treatment with EGCG. **a** Quantification of mean normalized area of NOX activation as measured from τ_2_ (enzyme-bound) NAD(P)H fluorescence lifetime images of MACS-purified splenic CD11b^+^ monocytes from healthy controls (*n* = 4), from mice immunized only with Freund’s Complete Adjuvant (CFA, *n* = 4) and from mice with EAE—at onset of disease (*n* = 3), at peak of disease (*n* = 5) or treated with EGCG (*n* = 4) encompassing two to four independent EAE experiments. **b** Quantification of mean normalized area of NOX activation of MACS-enriched blood CD11b^+^ human monocytes from age- and gender-matched healthy controls (*n* = 6), patients with clinically isolated syndrome (CIS, *n* = 5), untreated RRMS patients (*n* = 6), RRMS patients treated either with glatiramer acetate (GA) and placebo (*n* = 6) or with glatiramer acetate and EGCG (*n* = 6) as well as SPMS patients with their regular MS-related therapy (*n* = 6). **a**, **b** Data points are mean values of at least 50 cells from one individual, for the statistic evaluation we applied ANOVA tests (*p < 0.05, **p < 0.01, ***p < 0.001). **c** Representative color-encoded τ_2_ NAD(P)H fluorescence lifetime images of MACS-enriched blood CD11b^+^ human monocytes

To translate our findings to MS, we isolated CD11b^+^ monocytes from peripheral blood mononuclear cells (PBMC) taken from MS patients, at different stages of the disease, including CIS, RRMS and SPMS. In healthy humans and healthy mice, CD11b^+^ monocytes were not activated after isolation, i.e., the area of NOX activation amounted to 3.4 ± 0.6 and 3.4 ± 1.9 %, respectively. In untreated RRMS patients, the area of NOX activation was 18.3 ± 2.5 %, in mice at peak of EAE, the NOX activation area was 18.5 ± 3.5 %. The high levels of NOX activation in peripheral CD11b^+^ cells of mice with EAE and of untreated RRMS patients could be ameliorated by treatment with glatiramer acetate (GA) alone, to only 10.8 ± 2.8 % (in RRMS patients). Upon combination treatment with both GA and additional systemic administration of EGCG, levels were even further reduced, almost to the levels of healthy controls: 5.8 ± 3.4 % in mice and 6.4 ± 1.2 % in RRMS patients (Fig. 5).

To understand the kinetics of NOX activation leading to oxidative stress during the course of the disease, we analyzed PBMCs of newly diagnosed (untreated) patients with CIS, untreated RRMS patients and differently treated SPMS patients. We observed little NOX activation in CIS patients (3.6 ± 1.2 %), and markedly elevated NOX activation in untreated RRMS patients (18.3 ± 2.5 %) and in treated SPMS patients (15.1 ± 3.2 %) (Fig. 5b). These data could be reproduced in mice, since at onset of disease CD11b^+^ splenocytes showed almost normal NOX activation (5.0 ± 1.1 % %), while at peak of disease the values were similar to those measured in untreated RRMS patients (18.5 ± 3.5 %). Immunization with adjuvant only (CFA without MOG) did not increase NOX activation (3.0 ± 0.9 %) in CD11b^+^ splenocytes. All values of NOX activation area represent mean values acquired over 30–120 cells per individual.

**Fig. 6:**
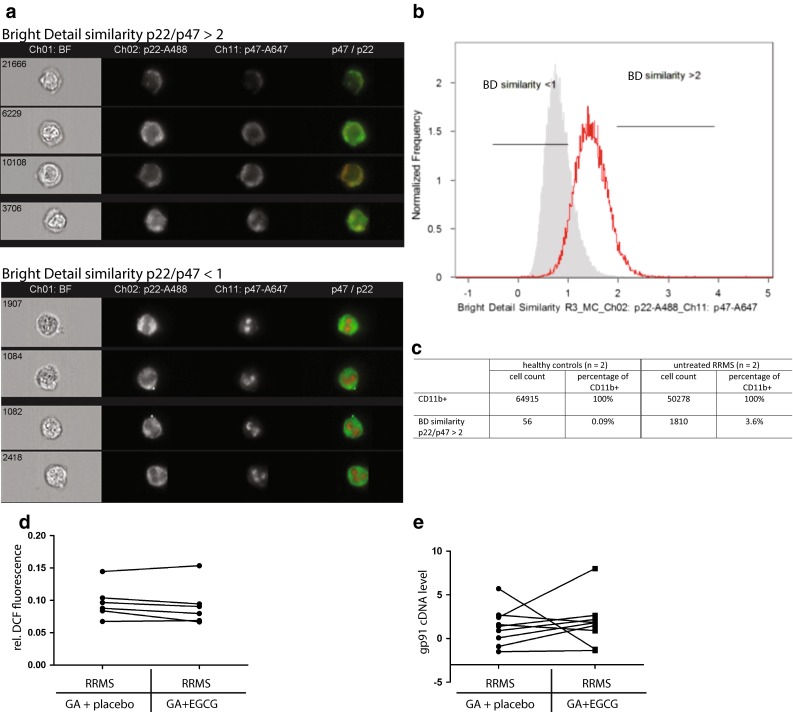
NOX subunits expression levels and co-localization in human peripheral CD11b^+^ cells as well as ROS detection in serum during chronic neuroinflammation. **a** Representative images of cells, generated by imaging flow cytometry immunostained for CD11b, p22 (membrane-bound NOX subunit) and p47 (cytosolic NOX subunit). Cells shown are gated on CD11b (not shown). The upper panel depicts cells with high bright detail similarity (>2) between p22 and p47 patterns (activated NOX), whereas the lower panel shows cells with low bright detail similarity (<1). **b** Less CD11b^+^ cells of a healthy control show high similarity (>2) between p22 and p47 as compared to an untreated RRMS patient. **c** The table depicts corresponding cell numbers and percentages from two healthy controls and two untreated RRMS patients showing that CD11b^+^ cells with high similarity between p22 and p47, indicating activated NOX, are at least 20× more frequent in untreated RRMS patients than in healthy controls. **d** Results of DCF fluorescence-based ROS/RNS detection assay in serum of patients show a general trend of lower ROS/RNS production in glatiramer acetate (GA) treated RRMS patients after EGCG complementary therapy as compared to placebo. **e** Analyzed gp91 cDNA levels of PBMCs with qRT-PCR showed no difference in expression levels in patients pre- or post-treatment with EGCG

To address if EGCG counteracts a possible overexpression of NOX2 subunits in MS, rather than direct NOX2 overactivation, we performed qRT-PCR analysis of PBMCs from ECGC-treated and untreated patients. We did not detect any differences in the expression level of the gp91 (NOX2 subunit) in patients treated with GA and placebo as compared to treatment with GA and EGCG (Fig. 6e), indicating that the reduced activation of NOX2 in EGCG-treated patients was not caused by reduced gp91 gene expression. Rather, this observation could be explained by direct inhibition of NOX2 activation by EGCG, which is in accordance with the fact that EGCG competes with NADPH in binding to the NADPH-binding site of enzymes [6] including the NADPH oxidases (NOX enzymes). In vitro data from our group using 100 µM EGCG yielded similar results, as they lead to an immediate shift with more free NAD(P)H with a shorter fluorescence lifetime (data not shown).

As NOX2 activity requires the fusion of the membrane-bound p22 and cytosolic p47 components, we examined the co-localization of p22 and p47 in human CD11b^+^ PBMCs by imaging cytometry to confirm our FLIM data with an independent approach. The co-localization was quantified as the Bright Detail Similarity (BDS), which measures the correlation coefficient between the intensities in localized bright spots of radius 1 µm or less. In healthy donors, less than 0.1 % of CD11b^+^ cells demonstrated co-localization of the p22 and p47 subunits, as indicated by a BDS >2, while RRMS patients had a markedly increased proportion of cells in which the two subunits co-localized, with 3.6 % of CD11b^+^ cells having a BDS >2 (Fig. 6a–c). Using a DCF (Di-Chloridium Fluorescein) fluorescence-based ROS/RNS detection assay in serum, we measured a general trend towards lower ROS/RNS concentrations in GA-treated RRMS patients with EGCG co-therapy as compared to placebo (Fig. 6d). Consistent with MS pathology (with the brain as the target of inflammation) and the frequencies of overactivated cells in the blood as detected with imaging cytometry, the ROS/RNS are highly diluted in serum, and were not significantly different between the groups under investigation.

## Discussion

To elucidate how oxidative stress causes neuronal degeneration and to clarify the mechanisms of anti-oxidant therapy, it is necessary to have a tool which allows real-time monitoring of functional changes at cellular and sub-cellular level. Applying this tool both within the central nervous system—where the damage takes place—and in the peripheral immune system demonstrates novel links between human disease and animal models, and offers unique functional insights into the systemic impact of MS.

In the present study, we monitored for the first time the functionality of NADPH oxidases catalyzing the production of ROS, which leads to oxidative stress in vivo. The fact that NOX enzymes are membrane-bound enzymes facilitates the identification of their cellular origins. Our intravital imaging and marker-free NAD(P)H-FLIM data demonstrate that NADPH oxidases are overactivated in astrocytes (sulforh101 labeled) during neuroinflammation, in addition to the known contributors, LysM^+^ and CX_3_CR_1_^+^ phagocytes, previously reported and recently corroborated in bone marrow chimeric mice deficient for NRROS, a negative regulator of ROS [38, 42]. Our intravital data regarding the role of astrocytes in chronic neuroinflammation are supported by histological studies showing that Noxo1 is expressed in GFAP^+^ cells, and that negative p22 immunostaining [45] in astrocytes does not exclude a NOX2-independent functional NADPH oxidases activation in vivo [54]. Additionally, we showed that NOX enzymes activation was spatially associated with lesion sites in EAE, and correlated with locations of neuronal dysfunction. This finding is consistent with the report that mice with knockout of the common subunit of NOX enzymes (p47^−/−^ mice) are more resistant to EAE [57] supporting the idea that NOX2, the phagocytic isotype, is not the only member of the NOX family that may contribute to excessive ROS production in chronic neuroinflammation. In line with this, mice lacking only NOX2 (gp91^−/−^) develop only a slightly milder form of EAE [29] and expression levels of NOX enzyme subunits other than NOX2 were elevated within active MS lesions [13]. One additional candidate is NOX1, which was reported to be neurotoxic in microglia [8]. Importantly, our NAD(P)H-FLIM technique to detect the catalysis of oxidative stress does not discriminate between NOX isotypes. Rather, it detects their increased activation as compared to other NAD(P)H-dependent enzymes responsible for basic cellular processes that are present in the same observation volume (0.4 × 0.4 × 1.5 µm^3^). Therefore, the hallmark of our technique is its capacity to monitor excessive NOX enzyme function as a reliable indicator of oxidative stress, rather than dissecting the activation of specific NOX isotypes.

NADPH oxidases activation was not restricted to a specific cell type, and was not preferentially found co-localized with the major compartments of the immune system (macrophages/activated microglia) and of the CNS (neurons), suggesting a predominant contribution of locally acting soluble factors like TNF-α or glutamate [15, 62]. Glutamate is not only an excitotoxic factor but was also shown to induce NOX activation. Supporting this interpretation, we found that local treatment with glutamate caused a significant increase of the NOX enzymes activation area as revealed by intravital NAD(P)H-FLIM in the brain stem of healthy mice (Suppl. Fig. 5).

As macrophages/microglia are two of the main producers of ROS in EAE, it was unexpected that only up to 50 % of the area of macrophages/microglia (LysM^+^tdRFP cells and CX_3_CR_1_^+/−^EGFP cells) showed activation of NOX enzymes in vivo (Fig. 4e). This is consistent with the findings that the reactive oxidative burst is counter-regulated in MS by myelin-loaded macrophages up-regulating anti-oxidant enzymes [14, 17, 58]. The toxic role of excessive ROS production in MS pathogenesis has been proposed to be related to multiple mechanisms, including enhanced blood–brain barrier transmigration [56], oligodendrocyte damage, and mitochondrial damage leading to further ROS production. Here, we provide evidence for an additional mechanism of NOX enzymes-mediated pathogenesis in MS: direct induction of neuronal dysfunction. We demonstrated in vivo that NOX enzymes activity leading to excessive ROS production was spatially correlated with pathologically increased neuronal calcium—a reliable indicator of neuronal dysfunction at lesion sites [7, 48].

The activation of astrocytes towards oxidative stress generation in chronic neuroinflammation supports the hypothesis that these cells are partially responsible for the accumulated neuronal dysfunction and damage over the course of the disease. In the later phases of the disease, activated astrocytes and microglia may continue to enhance neuronal loss even in the absence of overt immune infiltration of the CNS. Thus, these cells may become a major target of therapy, especially in the progressive phase of the disease. We also employed our NAD(P)H-FLIM technique, under in vitro conditions, to monitor NOX enzymes activation in peripheral cells. The elevated NOX activation in monocytes from the periphery of EAE mice and of MS patients at various stages of the disease strengthens the view that both MS and EAE have a complex systemic dimension. Increased NOX activation in peripheral monocytes is not specific for MS, as we could also see moderately elevated NOX activation in individuals with common cold (data not shown). However, we saw a significant increase of NOX activation with disease progression, similar to our observation in the target organ, the CNS. The time-dependent increase in MS patients from no overactivation at the beginning of the disease (CIS patients and EAE onset) to elevated NOX activity in RRMS and SPMS patients’ PBMCs we observed, in conjunction with data from other groups showing overexpression of NOX subunits in the CNS tissue of progressive MS patients at lesion sites [13] supports the hypothesis that during the course of the disease the immune system (and possibly also CNS components like astrocytes and microglia) develops an “oxidative stress memory”. Our findings are furthermore in accordance with data showing that in the chronic phase of the disease oxidized DNA, damaged lipids, and proteins are present in PBMCs of MS patients [21], supporting the proposition that excessive ROS plays a major role in the progressive phase of the disease.

Collectively, these results point to new treatment strategies aiming to reduce oxidative stress in MS. EGCG, the major polyphenolic compound of the green tea plant has been investigated under numerous conditions. Although the molecular mechanisms of its actions are not fully understood [33], there is experimental data pointing to the inhibition of ROS production through EGCG in vitro [63]. Empirically, we showed that EGCG is neuroprotective in EAE, it is known to influence T cell and neuronal function [2, 19, 61] and clinical trials investigating the effects of EGCG (NCT00525668, NCT00799890 and NCT01417312) are ongoing.

In the current study, we demonstrated a direct anti-oxidative mechanism of EGCG on PBMCs of MS patients. Oral treatment with EGCG completely counteracted the chronic NOX overactivation in MS patients, consistent with a higher enzymatic activity, as we could not detect overexpression of NOX2 subunit gp91 by qRT-PCR. Since it is known that EGCG inhibits the binding of NADPH to enzymes within the cell, we observed the mechanism of blocking NADPH binding to NADPH oxidases.

Beside insights into the cellular sources of oxidative stress in EAE and MS, in the CNS and in the periphery, this work demonstrates the unique versatility of intravital NAD(P)H-FLIM as a marker-free method to investigate mechanisms of oxidative stress in inflammatory pathologies, underscoring its intriguing potential for biomedicine as well as clinical research, in a more general context.

## Electronic supplementary material

Supplementary material 1 (DOCX 82 kb)

Supplementary material 2 (PDF 8052 kb)

Supplementary material 3 (PDF 710 kb)

Supplementary material 4 (PDF 578 kb)

Supplementary material 5 (PDF 930 kb)

Supplementary material 6 (PDF 2393 kb)

Supplementary material 7 (PDF 5653 kb)

Supplementary material 8 (PDF 685 kb)

Supplementary material 9 (MOV 5742 kb)
