# Supplementary material for: Tracking CNS and systemic sources of oxidative stress during the course of chronic neuroinflammation
Source: Acta Neuropathol. 2015 Oct 31;130(6):799–814. doi: 10.1007/s00401-015-1497-x (PMC4654749; doi:10.1007/s00401-015-1497-x)
Supplement: Supplementary file 1 — Supplementary material 1 (DOCX 82 kb) [file 401_2015_1497_MOESM1_ESM.docx]

Tracking CNS and systemic sources of oxidative stress during the course of chronic neuroinflammation

**- SUPPLEMENTAL MATERIAL -**

1. NAD(P)H-FLIM and selective detection of enzyme catalytic activity
2. FLIM data analysis
3. Supplemental methods
4. Two-photon laser-scanning microscope (TPLSM)
5. Mice
6. Experimental autoimmune encephalomyelitis (EAE)
7. FACS analysis
8. Imaging cytometric analysis of p22 and p47 localization in CD11b^+^ cells
9. Histology
10. Preparation of astrocytes/microglia co-cultures
11. Fluorescence-based ROS/RNS detection
12. Supplementary movie: Interaction of LysM^+^ tdRFP cells with neurons in the brain stem of *CerTN L15 x LysM tdRFP* mice affected by EAE
13. Supplementary figure legends and tables

## I. NAD(P)H-FLIM and selective enzyme detection

As previously described, the coenzymes nicotinamide adenosine dinucleotide (NADH) and nicotinamide adenosine dinucleotide phosphate (NADPH), hereafter NAD(P)H, are selectively detected both in cell culture[^1^](#_ENREF_1)^,^[^2^](#_ENREF_2) and in tissue by two-photon microscopy when they are excited at 760 nm and their fluorescence is detected at 460 ± 30 nm[^3^](#_ENREF_3).

The bi-exponential evaluation of time-resolved fluorescence imaging data acquired under these conditions using the parallelized TCSPC detector revealed, as expected, a short fluorescence lifetime τ_1_ of free NAD(P)H (~450 ps) and a longer fluorescence lifetime τ_2_ of enzyme-bound NAD(P)H, i.e. NAD(P)H involved in biochemical reactions within the cell, catalyzed by the enzymes to which it is bound[^4^](#_ENREF_4). The short fluorescence lifetime τ_1_ is the average over all conformational structures of NAD(P)H[^5^](#_ENREF_5) (*Suppl. Fig. 3*). The fluorescence lifetime τ_2_ of the enzyme-bound NAD(P)H is strongly dependent on the NAD(P)H binding-site on the enzyme as measured under extra-cellular conditions in mixtures of NAD(P)H, various enzymes and corresponding substrates (*Suppl. Fig. 3*) and as reflected by typical histograms of the τ_2_-values in images of astrocytes in cell culture (*Suppl. Fig. 4*). Furthermore, the differences between the fluorescence lifetimes of enzyme-bound NAD(P)H are related both to enzyme as well as to the coenzyme itself, as recently reported under intracellular conditions by Blacker et al[^2^](#_ENREF_2). We could measure extracellularly that sorbitol dehydrogenase binding to NADH in the presence of sorbitol leads to a fluorescence lifetime of 2200 ps while when binding to NADPH leads to a fluorescence lifetime of 2000 ps. The histogram of un-stimulated astrocytes can be best approximated by multi-peak Gaussian function, revealing the presence of two major types of binding sites on NAD(P)H-dependent enzymes in the normally functional cells. Moreover, the τ_2_-histograms slightly vary from cell type to cell type revealing a slightly different basal enzyme activity within different cell subsets (e.g. astrocytes vs. peritoneal macrophages or CD11b+ peripheral blood cells).

Activation of NADPH oxidases (NOX1-4, DUOX1,2) using a 10 nM solution of phorbol-myristrate acetate (PMA, Sigma Aldrich, Germany) in the corresponding cell medium over 30 min. or *Staphylococcus aureus* coated polystyrene beads (0.02 % mass concentration, *pH*rodo beads, Life Technologies, Germany) in various cells types including macrophages, microglia and astrocytes led to an additional peak in the fluorescence lifetime histogram of the enzyme-bound NAD(P)H lifetime map (*Suppl. Fig. 4*). This effect could be counteracted by the addition of an inhibitor of NADPH oxidase, i.e. 100 µM 4-(2-aminoethyl) benzenesulfonyl fluoride hydrochloride (AEBSF) solution in PBS[^6^](#_ENREF_6) (*Suppl. Fig. 4*)., and did not appear during unspecific metabolic stimulation with 20 mM glucose (*Suppl. Fig. 4*). AEBSF blocks the assembly of NOX by blocking the binding of the proline-rich domain on p47 to the SH3 domain of p22. It inhibits the activity of proteases, but is not known to interfere with the function of other NAD(P)H-dependent enzymes. Based on this additional peak in the τ_2_-histogram, we set the gate of activated NOX between 3.3 and 3.9 ns – the “NOX only” gate. We extract from the complete image just the pixels characterized by a τ_2_ in this gate, retaining the information about their location within the image and, thus, within the sample (*Suppl. Fig. 4*). By using a rectangular gate we exclude the observation volumes (pixels) in which NOX is not abundantly bound to NADPH as compared to the binding of other enzymes either to NADH or to NADPH. In this case, the use of Gaussian gates would increase the sensitivity of our method in detecting NOX activation leading to oxidative stress.

The typical fluorescence lifetime of approx. 3.65 ns for NADPH coupled to NOX in the case of oxidative stress as measured by us under different conditions was also observed by Blacker et al. In this case, the longer fluorescence lifetime is associated with the NADPH bound to enzymes[^2^](#_ENREF_2). Since NOX (NADPH and not NADH binding enzymes) are highly involved in oxidative stress, their data strongly support our observations.

Additionally, adding NaCN to cells previously treated with PMA to activate NOX enzymes and induce ROS production leads to a complete loss of fluorescence with a lifetime over 1000 ps, i.e. the fluorescence lifetime of enzyme-bound NAD(P)H. This indicates that in our case the fluorescence signal observed by FLIM under oxidative stress conditions originates solely from NAD(P)H and not from other components like oxidized lipids as suggested by others [^7^](#_ENREF_7).

## II. FLIM data analysis

The effect of the instrument response function (IRF) of the p-TCSPC setup has been considered for the non-linear evaluation, i.e. re-convolution with the IRF was performed. Due to the fact that the IRF of the FLIM detector is symmetric, the benefit of re-convolution falls within the error margins. We used an iterative Levenberg-Marquardt algorithm to approximate the NAD(P)H-fluorescence decay data with a bi-exponential function – one exponential term corresponding to the free NAD(P)H, the other to the enzyme-bound NAD(P)H – for more robustness, we also used mono-exponential approximation of the second part of the fluorescence decay. In the whole manuscript, we display and focus only on the fluorescence lifetime image τ_2_ of the enzyme-bound NAD(P)H, namely the τ_2_ map, corresponding to the second exponential term in our fluorescence decay model.

In order to exclude numerical artifacts in the NAD(P)H-FLIM data analysis, we additionally evaluated the data using a model-free approach – the phasor approach[^8^](#_ENREF_8). This approach calculates the normalized Fourier transform of the fluorescence decay *I*(*t*) at a fixed virtual modulation frequency leading to a complex value. In a graph displaying the imaginary versus real part of these values, all the data are confined within a semicircle between 0 and 0.5 on the ordinate and 0 and 1 on the abscissa. If *I*(*t*) is mono-exponential, the values fall directly on the semicircle. The position of the mono-exponential decay times on the semicircle depends on the chosen virtual modulation frequency. If *I(t)* is bi-exponential, the values are located on the segment connecting the mono-exponential terms, yielding the bi-exponential decay function. The nearer a value is to an end of the segment, the larger is the contribution of the respective mono-exponential term to *I*(*t*).

Our results obtained at virtual modulation frequencies of 0.08 GHz (laser repetition rate) and 0.32 GHz using the phasor approach indicate that under intravital conditions most of the NAD(P)H is enzyme-bound – nearby mono-exponential decay characterized by a long fluorescence lifetime - corresponding to the expected high metabolic activity in living organisms. Furthermore, the phasor histograms (imaginary vs. real part of the normalized Fourier transform of *I*(*t*)) show a similar picture as the τ_2_ histogram assessed by linear/non-linear approximation using the Levenberg-Marquadt algorithm (*Suppl. Fig. 6*).

The evaluation of the FRET-FLIM data acquired in *CerTN L15* mice was performed bi-linearly, assuming a fluorescence lifetime of 0.7 ns for the FRET-quenched Cerulean and 2.3 ns for the unquenched Cerulean, as previously described[^9^](#_ENREF_9). We display and evaluate in this case the FRET ratio: the normalized contribution of the FRET-quenched mono-exponential term to the total fluorescence signal. Additionally, we transformed the FRET ratio values into absolute calcium concentrations based on a calibration curve acquired under extracellular conditions for the construct *TN L15*, under consideration of the Strickler-Berg equation concerned with the dependence of fluorescence lifetimes on the refractive index ^[9](#_ENREF_9)^.

Image representation and 3D/4D image reconstruction was performed with Volocity 5.2 (Perkin Elmer, Germany) and with ImageJ/FIJI.

## III. Supplemental methods

### Two-photon laser-scanning microscope (TPLSM)

Experiments were performed using a specialized multi-photon laser-scanning microscope for fluorescence lifetime imaging (FLIM). In brief, the beam of a tuneable fs-pulsed Ti:Sa laser (wavelength range 700 – 1080 nm, 140 fs, 80 MHz, Cameleon Ultra II, Coherent, Dieburg, Germany) is scanned by two galvanometric mirrors and focused into the sample by an objective lens for deep-tissue imaging (20x dipping lens, NA 0.95, WD 2 mm – Olympus, Hamburg, Germany). The resulting fluorescence signal is detected and analyzed either by a parallelized 16 channel TCSPC point detector (p-TCSPC FLIM-X16, LaVision Biotec GmbH, Bielefeld, Germany) or photomultiplier tubes (Hamamatsu, Japan). The p-TCSPC device is based on parallel photon detection with multi-anode (16 channels) photomultiplier tubes (PMT) and on evaluation relying on time-to-digital converter (TDC) electronics. Thus, the electronic dead time of the device is only 5.5 ns. Spectral discrimination of the fluorescence signal was achieved by appropriate dichroic mirrors and interference filters. NADH and NADPH (NAD(P)H) were excited at 760 nm and detected through an interference filter at 460 ± 30 nm. The time step (bin) was 160 ps and the time window for measuring the fluorescence decay was 9 ns. Amplex Red® (ROS and RNS indicator) was excited at 910 nm and detected at 593 ± 20 nm. Cerulean (CFP derivative) in the neurons of *CerTN L15* mice was excited at 850 nm and detected through a dichroic mirror (cut off wavelength 506 nm) and an interference filter of 460 ± 30 nm. For FRET-FLIM of Cerulean the parameters chosen for TCSPC detection were time step (bin) 80 ps and time window to measure the fluorescence decay 9 ns. EGFP in the CX_3_CR_1_ microglia and sulforhodamine 101 mainly labelling astrocytes were simultaneously excited at 850 or 880 nm and detected through a dichroic mirror (cut off wavelength 560 nm) and interference filters of 525 ± 25 nm and 593 ± 20 nm, respectively. YFP in the CD4+ cells (T cells) and sulforhodamine 101 were simultaneously excited at 880 nm and detected through a dichroic mirror (cut off wavelength 560 nm) and interference filters of 525 ± 25 nm and 593 ± 20 nm, respectively.

In intravital experiments on *CerTN L15* x *LysM tdRFP* mice we employed dual near infrared/infrared excitation[^10^](#_ENREF_10). Therefore, in addition to the Ti:Sa laser tuned to 850 nm, we used an optical parametric oscillator (OPO, APE, Berlin, Germany) tuned to 1110 nm.

### Experimental autoimmune encephalomyelitis (EAE)

EAE was induced as previously described^[11](#_ENREF_11)^. Briefly, mice were immunized subcutaneously with 150 µg of MOG_35–55_ (Pepceuticals, UK) emulsified in CFA (BD Difco, Germany) and received 200 ng pertussis toxin (PTx, List Biological Laboratories, Inc.) intraperitoneally at the time of immunization and 48 h later. Intravital multi-photon microscopy was performed at different stages of the disease, i.e. onset (1-2 days after appearance of first clinical symptoms) and peak (3-7 days after appearance of first clinical symptoms). For the EGCG experiments, the treated mice were given 300μg EGCG (Sigma-Aldrich, Deisenhofen, Germany) dissolved in 0.9% NaCl, administered by oral gavage twice daily, for a period of 14 days.

### Preparation of the brain stem for intravital imaging

The brain stem was exposed by carefully removing the musculature above the dorsal neck area and removing the dura mater between the first cervical vertebra and the occipital skull bone. The head was inclined for access to deeper brainstem regions and the brain stem superfused with isotonic Ringer solution. Anaesthesia depth was controlled by continuous CO_2_ measurements of exhaled gas and recorded with a CI-240 Microcapnograph (Columbus Instruments, USA) and by an Einthoven three-lead electrocardiogram (ECG). In order to avoid strong breathing artefacts in the brainstem of anesthetized mice, the ECG signal was correlated to the respiration rate and used as an external trigger for the image acquisition software, which controls the hardware of the microscope setup. Thus, each fluorescence stack was recorded in the same tissue region at the same point in the respiratory cycle of the mouse^[12](#_ENREF_12)^.

### FACS analysis

To isolate cells from the brain and spinal cord of the mice, the tissue was homogenized after PBS perfusion and a percoll gradient was performed according to standard protocols with 25% and 75% stock istotonic percoll (GE Helthcare) and HBSS. Cells were blocked with antibodies to Fcγ receptors (DRFZ, clone 2.4G2) to avoid nonspecific staining, and were subsequently stained with FITC-labeled rat anti-CD45 (BioLegend), Cy5 (DRFZ) or Pacific Blue™ (BioLegend) labeled rat anti-CD11b, fixable Viability Dye eFluor®780 (eBioscience), and in some experiments with armenian hamster anti-CD11c-PE/Cy7 (BioLegend), rat anti-Ly6C Pacific Blue (BioLegend), mouse anti-CX3CR1-APC (BioLegend) and/or biotin labeled anti-Ly6G (BioLegend) detected by Streptavidin anti-biotinylated PeCP-eFluor®710 (eBioscience) according to standard procedures followed by fixation using 4% Paraformaldehyde (Electron Microscopy Science) for 10 minutes. FACS analysis was performed on a LSR Fortessa.

### Imaging cytometric analysis of P22 and P47 localization in CD11b^+^ cells

PBMCs were isolated as above for flow cytometry, blocked, stained for surface p22, fixed and permeabilized, and stained intra-cellularly for p47. Primary antibodies were detected with Alexa 488 and Alexa 647, respectively. Cells were acquired on an Amnis Imagestream Mark II with 405 nm, 488 nm, and 642 nm excitation, and 12 detection channels, using the 60x, NA 1.25 objective lens. A minimum of 20,000 focused CD11b+ single cells were collected for each sample, and the bright detail similarity R3 feature was calculated on all CD11b+ cells positive for both p22 and p47. Based on visual inspection of the cells, we set our threshold for high similarity to BDS > 2. Antibodies used were: CD11b-Pacific Blue (DRFZ), anti-rabbit anti-p22 (Santa Cruz Biotechnology, Dallas, TX, US), anti-goat anti-p47 (Abcam, Germany), donkey anti-rabbit IgG-Alexa 488 (Life Technologies, Germany), and donkey anti-goat-Alexa 647.

### histology

At the time of sacrifice, mice were transcardially perfused successively with PBS and 4% PFA and tissues removed for fixation in 4% PFA solution, as described previously^11^. Brainstems were cut embedded in Tissue Tek (Sakura), frozen in a methylbutane/dry ice mixture, and then cut into 4 μm sections with a cryostat. Sections were stained with rat anti-CD11b-Alexa®647 (DRFZ) and mouse anti-GFAP-Alexa®488 (eBioscience, Germany) respectively rabbit anti-Noxo-1 (Novus Biologicals, Germany) and detected by the second antibody donkey anti-rabbit Alexa®647 (Life Technologies, Germany) and mouse anti-GFAPAlexa®488 (eBioscience, Germany).

### Preparation of astrocytes/microglia co-cultures

New born mice were decapitated at days 1 to 3 after birth. Brains were isolated and meninges were removed carefully under a Zoom 2000 microscope. Mouse heads and isolated brains were stored in ice-cold PBS during the whole preparation procedure. Afterwards PBS was aspirated, and each mouse brain was covered with 1 ml of microglia culture medium and shredded slowly with a 5 ml-pipette, and subsequently with a Pasteur glass pipette (previously narrowed by scorching). Mashed brains were transferred to 75 cm² - cell culture flasks - one brain per flask -, supplied with 10 ml of microglia culture medium and incubated at 37°C/0.5 % CO_2_. After two days, a few astrocyte islands had adhered to the bottom of the flask. They were washed twice with PBS to remove cell debris and fed with 10 ml of fresh medium. After 9 to 12 days *in vitro*, when the astrocyte layer was confluent, medium was exchanged for 10 ml fresh microglia culture medium and 5 ml L929-conditioned medium, to enhance growth of microglia.

### Fluorescence-based ROS/RNS detection

For the analysis of relative concentrations of reactive oxygen species (ROS) and of reactive nitrogen species (RNS) in the serum we used a fluorescence-based in vitro ROS/RNS assay (OxiSelect, Cell Biolabs, CA, USA) according to the manufacturer’s protocol. Data represent relative fluorescent intensity of the ROS/RNS sensitive dye DCF (Di-Chloridium Fluoresceine) in serum samples.

## IV. Supplementary Movie 1

Interaction of LysM+ tdRFP cells with neurons in the brain stem of CerTN L15 x LysM tdRFP mice affected by EAE.

Time-lapse sequence of 3D fluorescence images with macrophages and activated microglia, i.e. LysM+ cells (red), invading the parenchyma in the brain stem of a *CerTN L15xLysM tdRFP* mouse affected by EAE at peak of disease (score 2.5). Neuronal structures (Thy1 expression cassette) are shown in green, while fiber-like reticular structures (made visible through the effect of second harmonic generation) are shown in blue. Dual NIR/IR excitation at λ_exc_ = 850 + 1110 nm, mean laser powers 7-10 mW, λ_emm_ = 460 ± 30 nm (blue), 525 ± 25 nm (green), 593 ± 20 nm (red). Each frame represents a 300x300x50 µm³ volume.

# V. Supplemental figure Legends

## Supplementary Figure 1:

## *Histological Characterization of lesions in the brain stem of intravitally imaged mice affected by EAE.*

The brain stem of a C57B6 mouse affected by EAE (peak), in which the astrocytes were labeled with sulforh 101 for intravital imaging, was longitudinally sectioned. Superficial serial sections (4 µm thick), depicting the region imaged by intravital microscopy, were analyzed histologically. Hematoxylin-Eosin (left) and Luxol-Fast-Blue-PAS (middle and left) staining showed immune cell infiltration and demyelination as typically seen in C57BL6 MOG35-55 induced EAE. Left and middle image – scale bar = 200 µm; right image – scale bar = 20 µm. Next to round cells, many macrophages were present, some with intracellular PAS positive myelin degradation products **(a)**. Immunofluorescent labeling with anti-CD11b (magenta) and anti-GFAP (green) antibodies highlighted the macrophage infiltrations and the associated characteristic astrogliosis at the lesion site. Scale bar = 200 µm **(b)**. Additionally, the brain stem of another intravitally imaged mouse (*CerTN L15 x LysM tdRFP,* peak) was transversally sectioned (the typical sectioning strategy for histological analysis) and stained with DAPI (green) and CD11b (magenta). The white rectangular frame indicates a lesion imaged by intravital microscopy .Scale bar = 500 µm **(c)**.

## Supplementary Figure 2:

Calibration and controls for murine imaging experiments.

In healthy controls of *CerTN L15* x *LysM tdRFP* mice almost no tdRFP positive cells (red) reside in the brain stem, i.e. most microglia do not express tdRFP. Scale bar = 50 µm, λ_exc_ = 850 nm and 1110 nm, laser power 3 mW, λ_emm_ = 525 ± 25 nm and 593 ± 20 nm. Scale bar = 50 µm, λ_exc_ = 850 nm, laser power 5 mW, λ_emm_ = 460 ± 30 nm (**a**). Amplex Red^®^ is an indicator of peroxide groups present in ROS and RNS, responsible for oxidative stress. The graph displays the calibration curve of the fluorescence intensity of a 1 µM Amplex Red^®^ solution in PBS, showing its dependence on the H_2_O_2_ concentration. λ_exc_ = 910 nm, mean laser power 5 mW, λ_emm_ = 593 ± 20 nm (**b**).

## Supplementary Figure 3:

Principle of NAD(P)H-FLIM under extra- and intracellular conditions.

The fluorescence lifetime histograms of NADH/NADPH - unbound as well as bound to various enzymes - as measured under extra-cellular conditions give insight into how NAD(P)H-FLIM can be used for enzyme fingerprinting (**a**). Representative fluorescence decay image acquired in isolated peritoneal macrophages are evaluated by a non-linear biexponential Levenberg-Marquadt algorithm or monoexponentially by excluding the first approx. 500 ps of the fluorescence decay (relevant for free NADH and NADPH). We extracted and further analyzed the fluorescence lifetime of the second term of the NAD(P)H fluorescence decay corresponding to the enzyme-bound NAD(P)H – τ_2_. λ_exc_ = 760 nm, mean laser power 7 mW, λ_emm_ = 460 ± 30 nm (**b**).

## Supplementary Figure 4:

Selective detection of NADPH oxidases (NOX enzymes) by NAD(P)H-FLIM.

The τ_2_-maps as well as the corresponding histogram of astrocytes (**a**) as an example of various cell types (monocytes, peritoneal macrophages, primary microglia, polymorphonuclear cells) change upon addition of phorbol-myristrate acetate (PMA) or *Staphylococcus aureus* coated polystyrene beads: an additional peak at longer lifetimes appears. Based on the fluorescence lifetime histograms, we set the gate for NADPH oxidase detection between 3.3 and 3.9 ns (**b**). AEBSF, an inhibitor of all NOX enzymes, counteracts the effect of PMA, so that fluorescence lifetimes in the range 3.3 to 3.9 ns could not be detected in macrophages. An unspecific stimulation with glucose led to the same result, i.e. no fluorescence lifetime peak in the τ_2_ histogram beyond 3.0 ns. λ_exc_ = 760 nm, mean laser power 3-7 mW, λ_emm_ = 460 ± 30 nm (**c**). Fluorescence decay curves and corresponding NAD(P)H fluorescence images and τ_2_-maps of B cells kept in growth medium, treated with 100 nM PMA and subsequently treated with NaCN as a NAD(P)H metabolism blocker (**d**).

## Supplementary Figure 5:

EAE and glutamate induces NOX Enzymes Activation in vivo.

Typical τ_2_ enzyme-bound NAD(P)H-FLIM images of brain stem tissue in healthy controls and in EAE (onset and peak of the disease) (**a**). The excitotoxic agent glutamate was applied onto the brainstem of healthy C57Bl/6 mice (n=2). The fluorescence lifetime of enzyme-bound NAD(P)H was measured before and 20 minutes after local application. Whereas in healthy controls there was little to no NOX activation detectable, after glutamate addition the percentage of NOX-activation area was significantly higher (**b**). Displayed is the mean of 3 (before) and 6 (after glutamate) different fields of view per mouse in (**c**). λ_exc_ = 760 nm, mean laser power 7-10 mW, λ_emm_ = 460 ± 30 nm. Scale bar 50 µm.

## Supplementary Figure 6:

Comparative non-linear bi-exponential approximation and phasor approach analysis of intravital NAD(P)H-FLIM data in EAE.

NAD(P)H fluorescence decay data were acquired within the brain stem in healthy controls and in mice affected by EAE, at peak of the disease. The evaluation using a Levenberg-Marquadt iterative, bi-exponential algorithm (**a** - peak EAE, **d** – healthy control) lead to the same location of NOX within tissue (longer fluorescence lifetime τ_2_ in red) as the phasor approach (EAE: (**b**) at 0.08 GHz and (**c**) at 0.32 GHz, healthy control: (**e**) at 0.08 GHz and (**f**) at 0.32 GHz). Both the real (upper images in b,c,e and f) and the imaginary (lower images in b,c,e and f) part maps of the Fourier-transformed fluorescence decay show the same trend. Additionally, the phasor histograms (**g**)at 0.08 GHz and (**h**) at 0.32 GHz) show that whereas in healthy controls the shorter fluorescence lifetime (2.3 ns) of metabolic enzymes dominates, in EAE the long fluorescence lifetime (3.65 ns) contribution becomes stronger and dominates the image within lesions, at peak of disease. λ_exc_ = 760 nm, mean laser power 7-10 mW, λ_emm_ = 460 ± 30 nm.

## Supplementary Figure 7:

FACS analysis – gating strategy.

(**a**) The CNS of LysM^+^tdRFP mice affected by EAE at peak of disease was analyzed by FACS to determine the overall overlap between the LysM^+^tdRFP^+^ and CX_3_CR_1_^+^ subsets. (**b**) We additionally investigated the characteristics of the LysM^+^tdRFP^+^ cells at peak EAE, within the CNS. Therefore we used the markers CD11b, CD45, Ly6C and Ly6G.

Table 1:

The cohorts chosen for this trial included healthy individuals, patients with relapsing-remitting MS (RRMS), secondary progressive MS (SPMS) and clinically isolated syndrome (CIS), age- and sex-matched. The clinical status was scored using the Expanded Disability Status Scale (EDSS) as standardized scoring system. GA = standard treatment with glatiramer acetate + placebo, GA+EGCG = treated with glatiramer acetate and additional epigallocatechin-3-gallate. The SPMS patients received various treatments (no GA).

| cohorts | sex  (f/m) | age (years) | disease duration (months) | EDSS | relapses in the last 12 months | NOX activation area (%) | | |
| --- | --- | --- | --- | --- | --- | --- | --- | --- |
|  |  |  |  |  |  | GA | GA+EGCG | No treatment |
| RRMS | f | 43 | 73 | 3.5 | 2 | 10.85 | 6.36 |  |
|  | f | 49 | 73 | 3.0 | 0 | 6.04 | 6.95 |  |
|  | f | 42 | 77 | 4.0 | 1 | 14.3 | 4.8 |  |
|  | f | 34 | 57 | 3.5 | 1 | 12.38 |  |  |
|  | f | 55 | 208 | 2.5 | 0 | 11.85 |  |  |
|  | m | 49 | 10 | 0 | 1 |  | 8.31 |  |
|  | f | 44 | 28 | 3.5 | 0 | 9.56 | 5.34 |  |
|  | f | 38 | 31 | 2.5 | 1 |  | 6.75 |  |
|  | f | 29 | 114 | 3.5 | 3 |  |  | 19.37 |
|  | f | 60 | 43 | 2.5 | 1 |  |  | 17.48 |
|  | f | 32 | 19 | 2.0 | 1 |  |  | 14.72 |
|  | m | 33 | 77 | 2.0 | 0 |  |  | 16.88 |
|  | f | 44 | 132 | 2.5 | 0 |  |  | 19.51 |
|  | f | 45 | 50 | 1.5 | 2 |  |  | 21.93 |
| CIS | m | 33 | 5 | 0 | 1 |  |  | 3.46 |
|  | f | 32 | 6 | 1.0 | 1 |  |  | 4.58 |
|  | f | 47 | 5 | 2.5 | 1 |  |  | 2.73 |
|  | f | 26 | 3 | 2.0 | 1 |  |  | 2.13 |
|  | f | 28 | 5 | 1.0 | 1 |  |  | 5.08 |
| healthy controls | m | 22 |  |  |  |  |  | 3.59 |
|  | f | 26 |  |  |  |  |  | 3.77 |
|  | m | 53 |  |  |  |  |  | 3.89 |
|  | f | 52 |  |  |  |  |  | 3.79 |
|  | m | 28 |  |  |  |  |  | 3.31 |
|  | f | 50 |  |  |  |  |  | 2.02 |

| cohort | sex  (f/m) | age (years) | disease duration (months) | EDSS | relapses in the last 12 months | NOX activation area (%) |
| --- | --- | --- | --- | --- | --- | --- |
| SPMS | m | 58 | 153 | 6.0 | 0 | 10.83 |
|  | f | 54 | 336 | 2.5 | 0 | 13.09 |
|  | m | 33 | 123 | 6.5 | 0 | 16.59 |
|  | m | 53 | 360 | 3.5 | 0 | 15.17 |
|  | m | 49 | 203 | 6.5 | 0 | 20.28 |
|  | f | 55 | 352 | 4.0 | 0 | 14.48 |

Table 2:

EAE experiment data and mouse strains included in this study, imaging time point with respect to immunization and disease phase are given. Each line represents one individual animal. The standardized scoring system for the clinical symptoms of EAE is as follows (0.0 – no signs, 1.0 – hind limb paresis, 2.0 – hind limb paralysis, 3.0 – hind limb paralysis and additional fore limb paresis, 4.0 – moribund, 5.0 - death).

| mouse strain | EAE score | days after imm. | EAE  phase | mean NOX activation area in % | s.d. |
| --- | --- | --- | --- | --- | --- |
| C57Bl6 | 0.75 | 12 | onset | 4.44 | 1.05 |
| C57Bl6 | 2.0 | 14 | peak | 12.44 | 4.81 |
| C57Bl6 | 2.5 | 15 | peak | 24.59 | 5.08 |
| C57Bl6 | 1.5 | 19 | peak | 19.08 | 4.90 |
| CerTN L15 x LysM tdRFP | 1.0 | 13 | peak | 13.20 | 1.06 |
| CerTN L15 x LysM tdRFP | 0.5 | 14 | onset | 4.7 | 0.42 |
| CerTN L15 x LysM tdRFP | 2.0 | 14 | peak | 10.65 | 0.51 |
| CerTN L15 x LysM tdRFP | 2.0 | 15 | peak | 7.18 | 1.42 |
| CerTN L15 x LysM tdRFP | 1.0 | 16 | onset | 7.23 | 0.57 |
| CerTN L15 x LysM tdRFP | 2.5 | 15 | peak | 16.99 | 9.02 |
| CerTN L15 x LysM tdRFP | 1.0 | 16 | onset | 3.94 | 2.48 |
| CX3CR1+/- EGFP | 1.5 | 13 | peak | 17.88 | 10.75 |
| CX3CR1+/- EGFP | 1.5 | 14 | peak | 11.61 | 1.34 |
| CX3CR1+/- EGFP | 1.0 | 15 | onset | 4.86 | 2.01 |
| CX3CR1+/- EGFP | 1.5 | 22 | peak | 8.56 | 1.60 |
| CX3CR1+/- EGFP | 2.0 | 21 | peak | 14.16 | 4.09 |
| CD4+ YFP | 2.0 | 13 | peak | 16.26 | 3.58 |
| CD4+ YFP | 2.0 | 15 | peak | 23.54 | 2.87 |

# References

1. Chance, B. Mitochondrial NADH redox state, monitoring discovery and deployment in tissue. *Methods in enzymology* **385**, 361-370 (2004).

2. Blacker, T.S.*, et al.* Separating NADH and NADPH fluorescence in live cells and tissues using FLIM. *Nature communications* **5**, 3936 (2014).

3. Konig, K. Multiphoton microscopy in life sciences. *J Microsc* **200**, 83-104 (2000).

4. Lakowicz, J.R., Szmacinski, H., Nowaczyk, K. & Johnson, M.L. Fluorescence lifetime imaging of free and protein-bound NADH. *Proceedings of the National Academy of Sciences of the United States of America* **89**, 1271-1275 (1992).

5. Vishwasrao, H.D., Heikal, A.A., Kasischke, K.A. & Webb, W.W. Conformational dependence of intracellular NADH on metabolic state revealed by associated fluorescence anisotropy. *The Journal of biological chemistry* **280**, 25119-25126 (2005).

6. Diatchuk, V., Lotan, O., Koshkin, V., Wikstroem, P. & Pick, E. Inhibition of NADPH oxidase activation by 4-(2-aminoethyl)-benzenesulfonyl fluoride and related compounds. *J Biol Chem* **272**, 13292-13301 (1997).

7. Datta, R., Alfonso-Garcia, A., Cinco, R. & Gratton, E. Fluorescence lifetime imaging of endogenous biomarker of oxidative stress. *Scientific reports* **5**, 9848 (2015).

8. Digman, M.A., Caiolfa, V.R., Zamai, M. & Gratton, E. The phasor approach to fluorescence lifetime imaging analysis. *Biophys J* **94**, L14-16 (2008).

9. Rinnenthal, J.L.*, et al.* Parallelized TCSPC for dynamic intravital fluorescence lifetime imaging: quantifying neuronal dysfunction in neuroinflammation. *PloS one* **8**, e60100 (2013).

10. Herz, J.*, et al.* Expanding two-photon intravital microscopy to the infrared by means of optical parametric oscillator. *Biophys J* **98**, 715-723 (2010).

11. Siffrin, V.*, et al.* In vivo imaging of partially reversible th17 cell-induced neuronal dysfunction in the course of encephalomyelitis. *Immunity* **33**, 424-436 (2010).

12. Niesner, R., Siffrin, V. & Zipp, F. Two-photon imaging of immune cells in neural tissue. *Cold Spring Harbor protocols* **2013**(2013).
