# Supplementary figures and images for: Tracking CNS and systemic sources of oxidative stress during the course of chronic neuroinflammation
Source: Acta Neuropathol. 2015 Oct 31;130(6):799–814. doi: 10.1007/s00401-015-1497-x (PMC4654749; doi:10.1007/s00401-015-1497-x)

Supplemental figure 1 - Mossakowski et al.

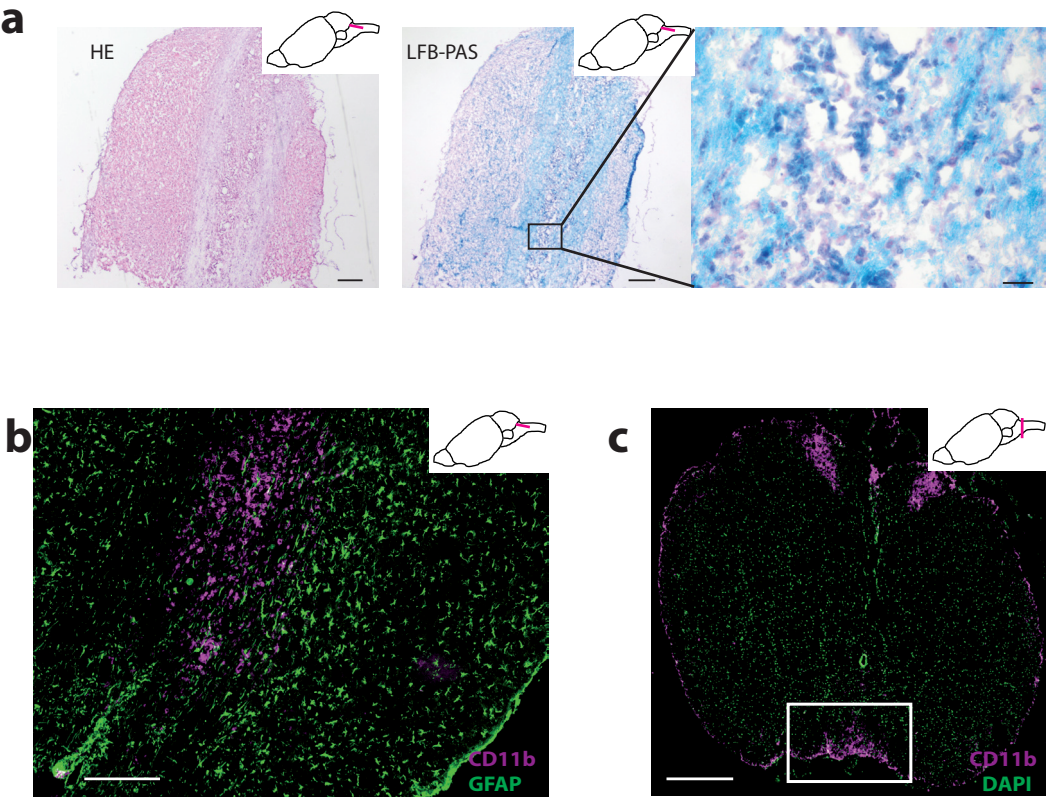

Supplement: Supplementary file 2 — Supplementary material 2 (PDF 8052 kb) [file 401_2015_1497_MOESM2_ESM.pdf]

Supplemental Figure 2 - Mossakowski et al.

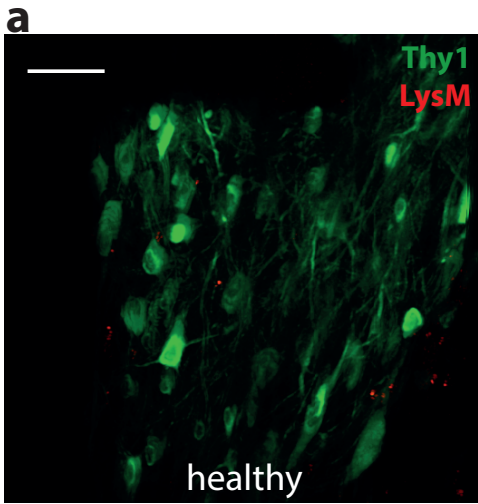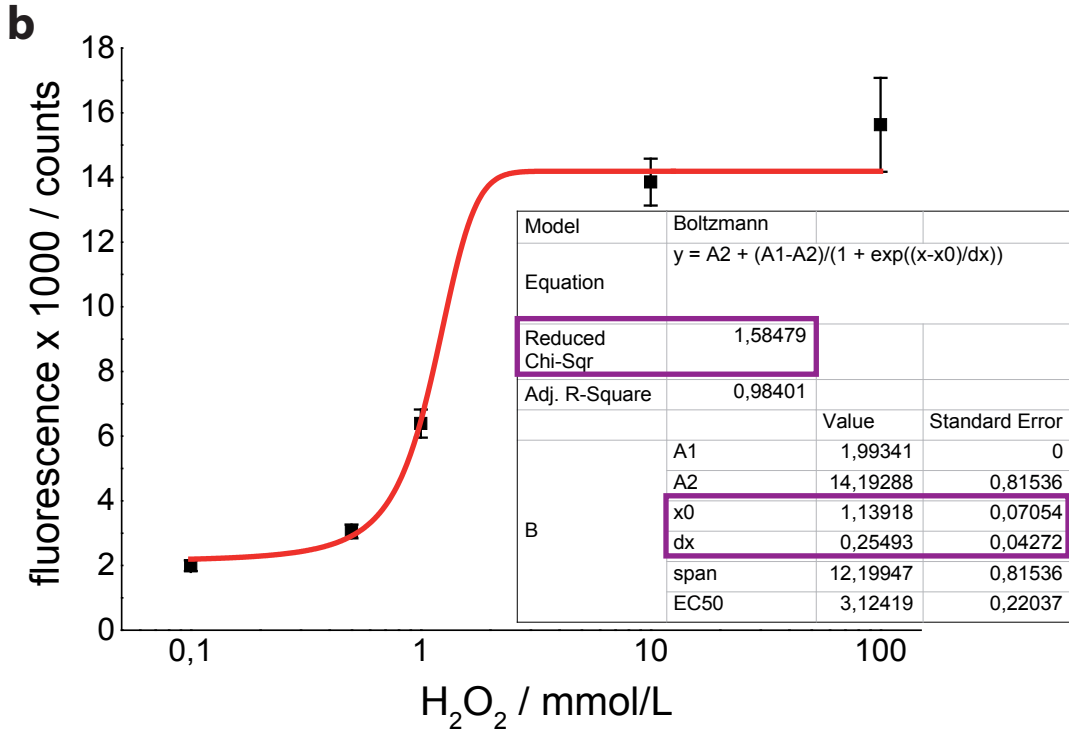

Supplement: Supplementary file 3 — Supplementary material 3 (PDF 710 kb) [file 401_2015_1497_MOESM3_ESM.pdf]

# Supplemental Figure 3 - Mossakowski et al.

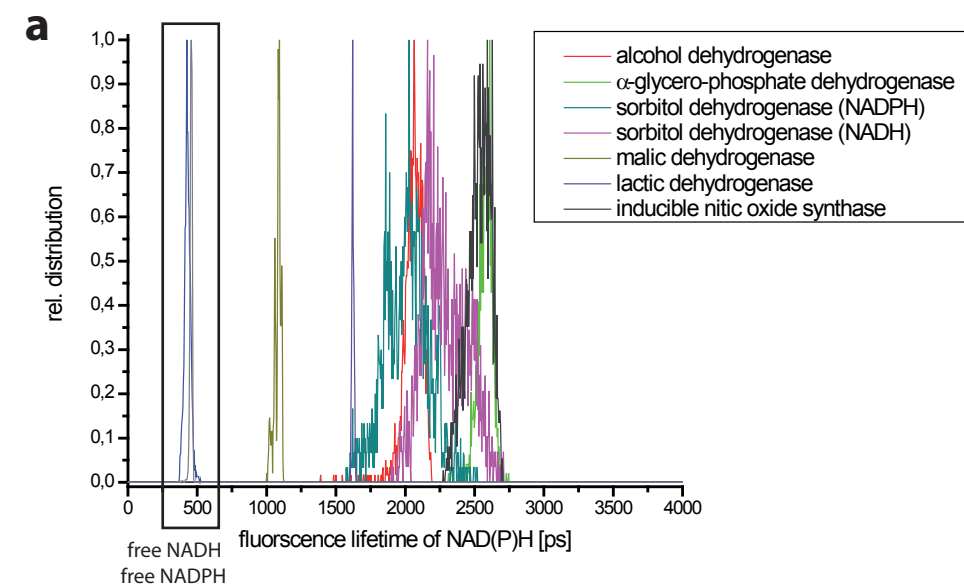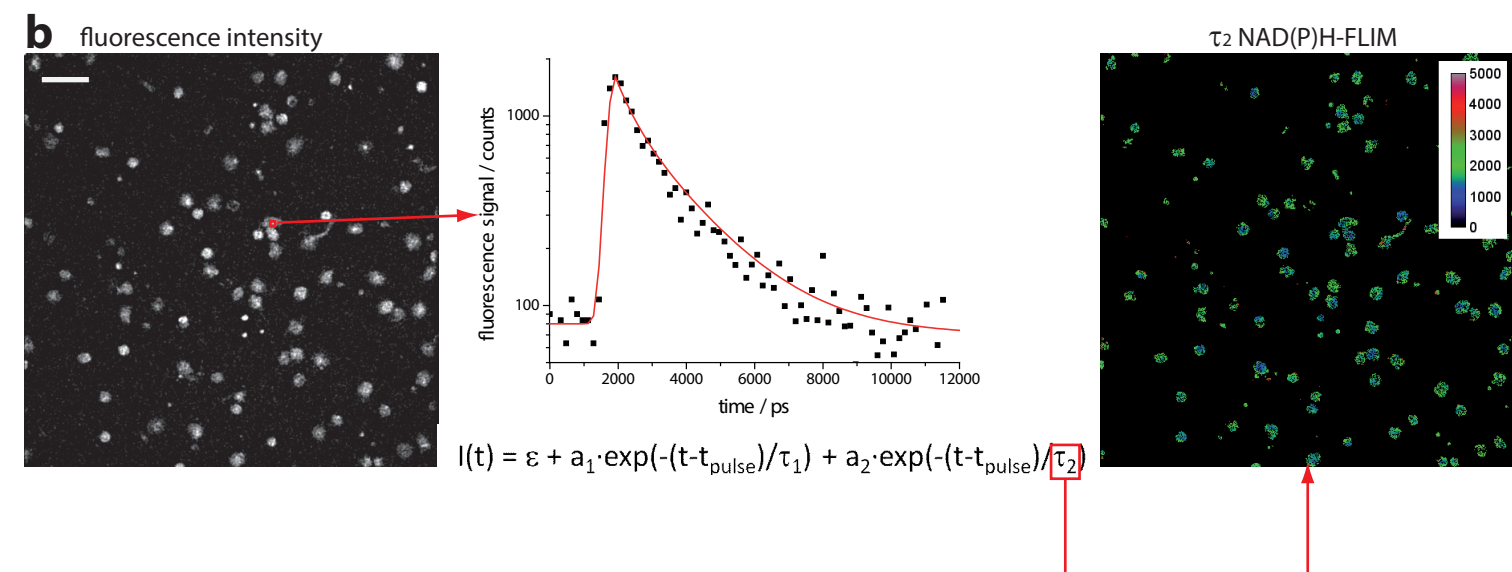

Supplement: Supplementary file 4 — Supplementary material 4 (PDF 578 kb) [file 401_2015_1497_MOESM4_ESM.pdf]

# Supplemental Figure 4 - Mossakowski et al.

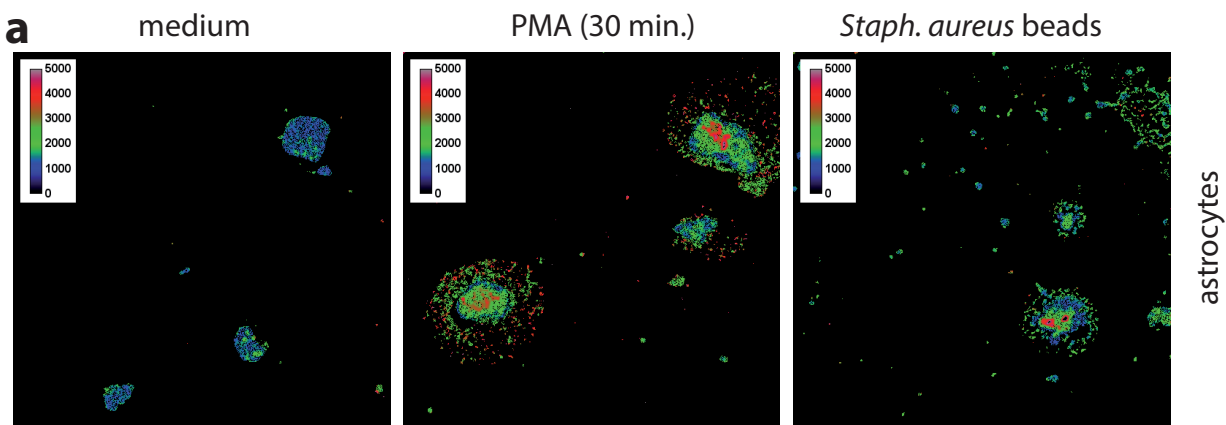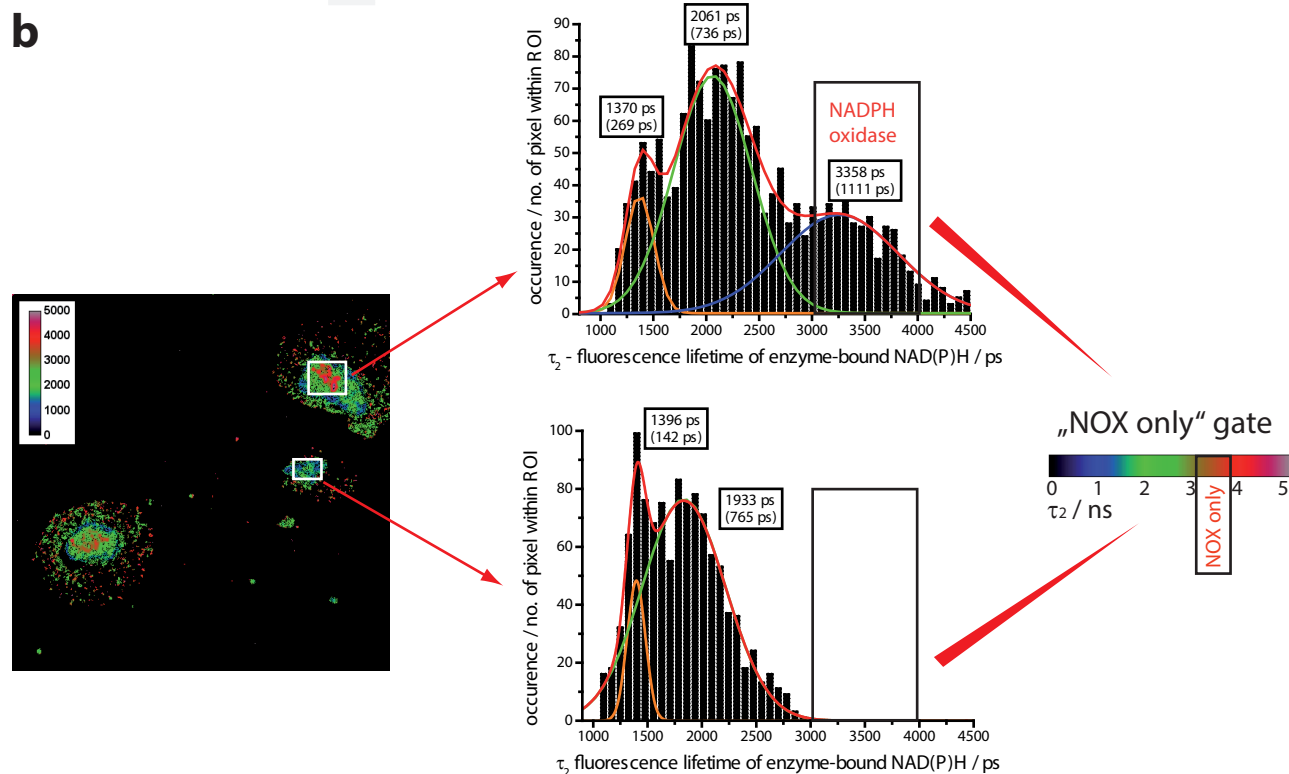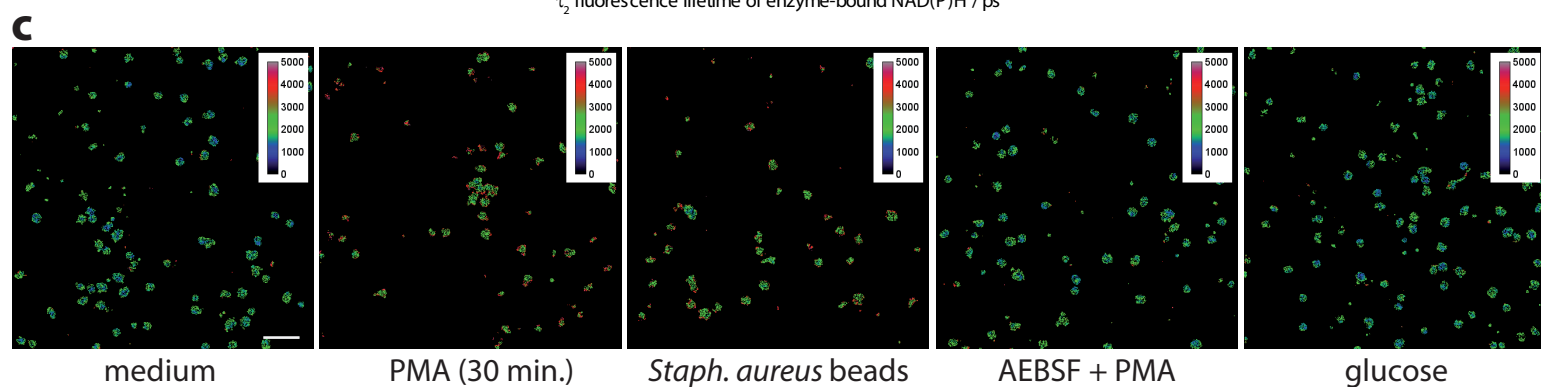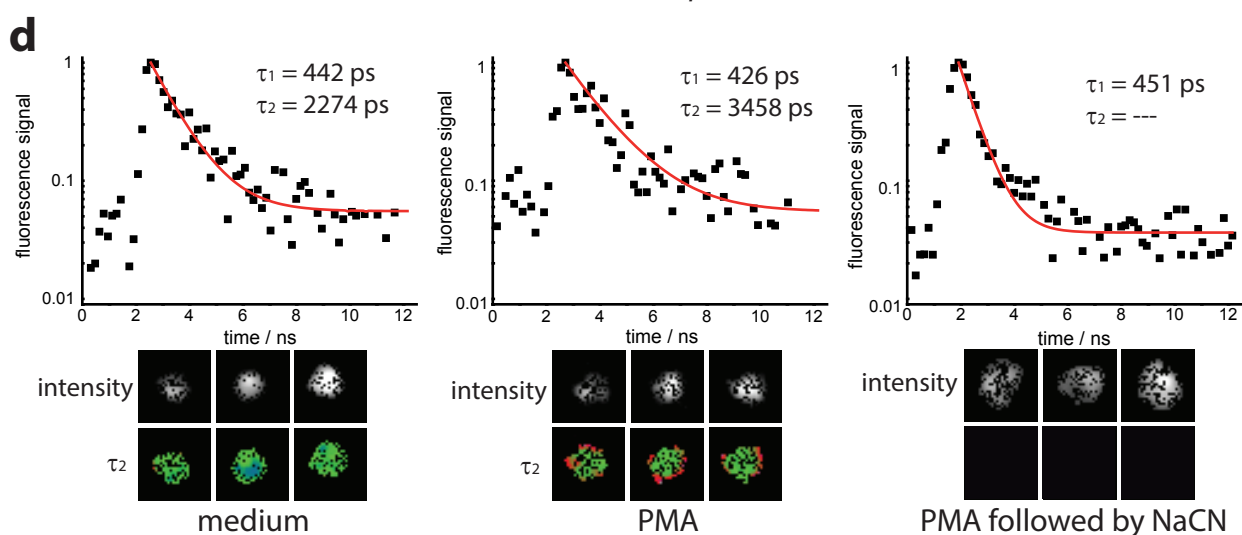

Supplement: Supplementary file 5 — Supplementary material 5 (PDF 930 kb) [file 401_2015_1497_MOESM5_ESM.pdf]

**a**

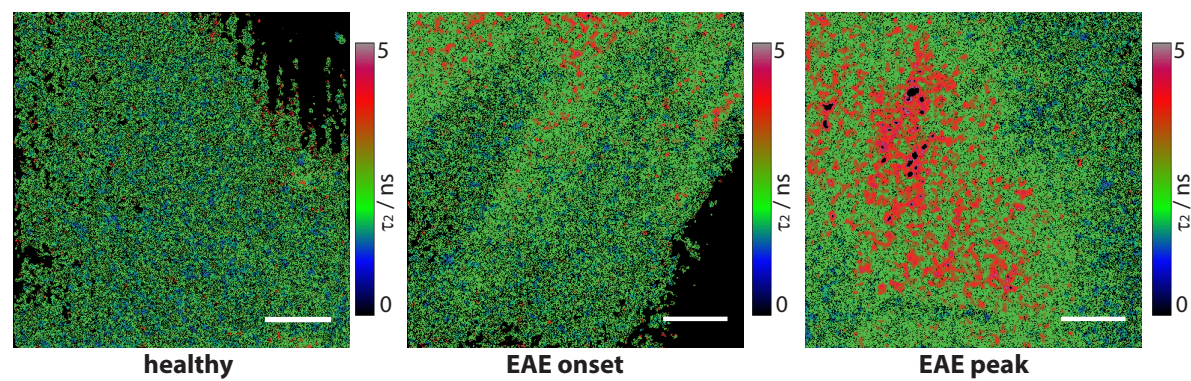

**b**

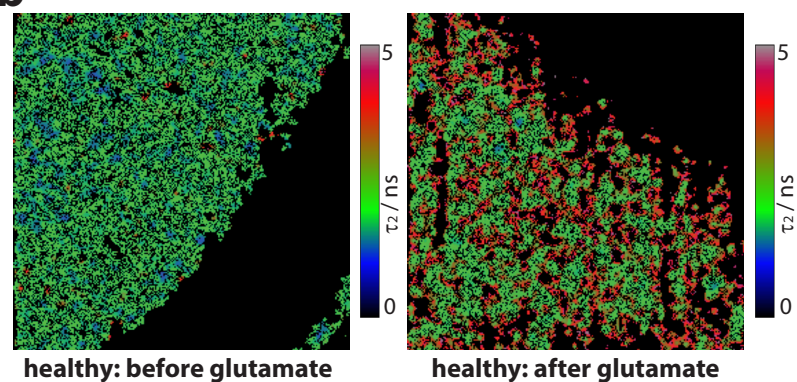

**c**

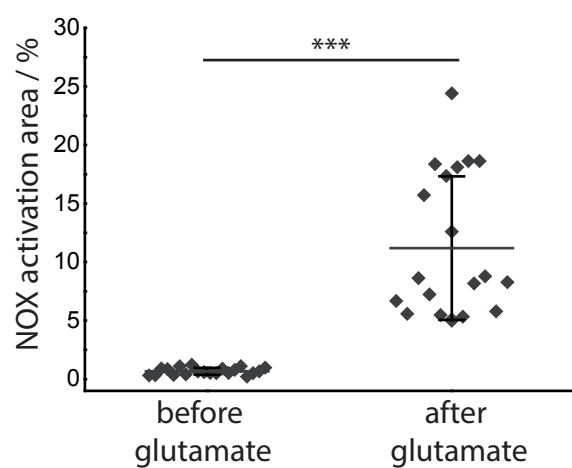

Supplement: Supplementary file 6 — Supplementary material 6 (PDF 2393 kb) [file 401_2015_1497_MOESM6_ESM.pdf]

Supplemental Figure 6 - Mossakowski et al.

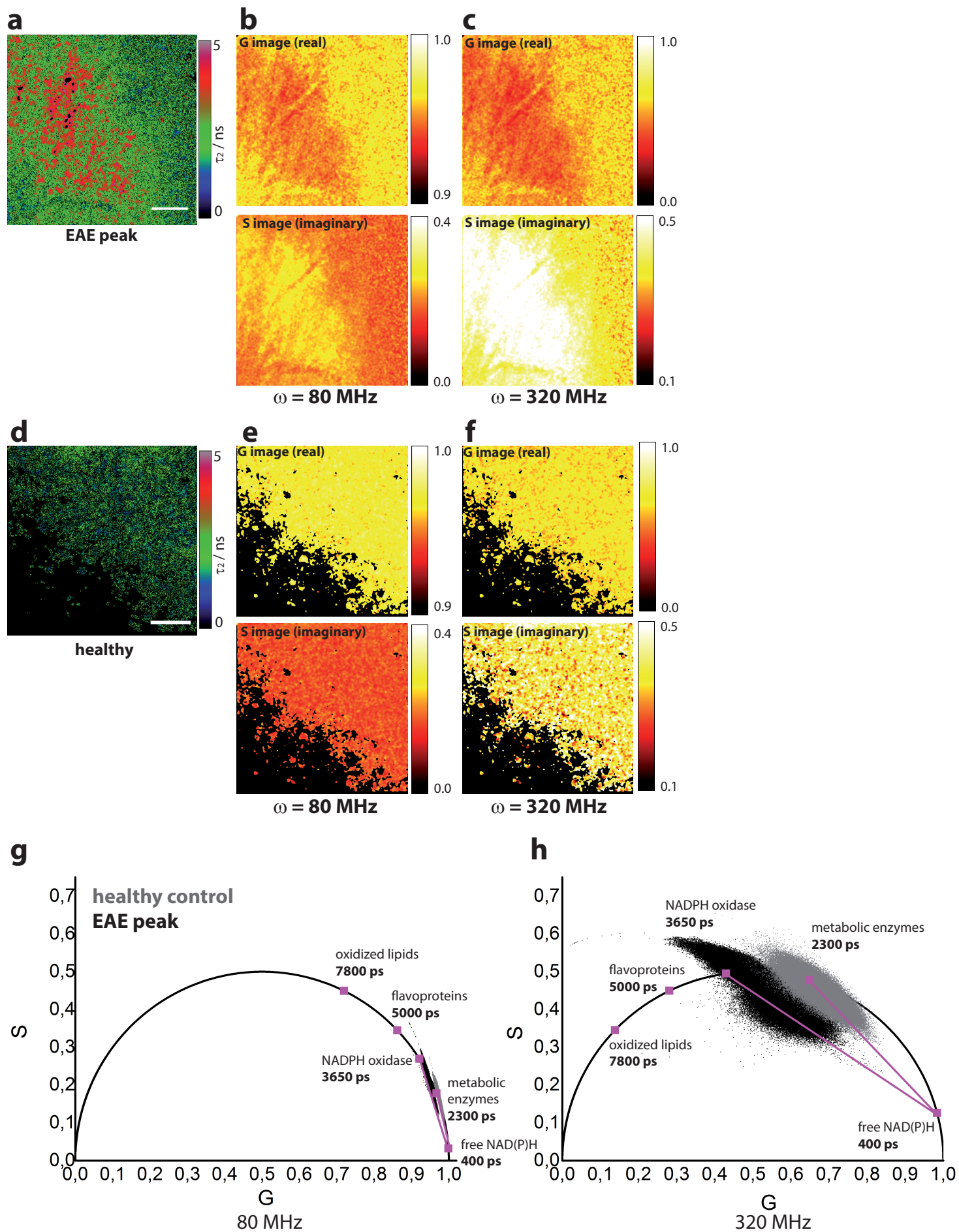

Supplement: Supplementary file 7 — Supplementary material 7 (PDF 5653 kb) [file 401_2015_1497_MOESM7_ESM.pdf]

# Supplemental Figure 7 - Mossakowski et al.

**a**

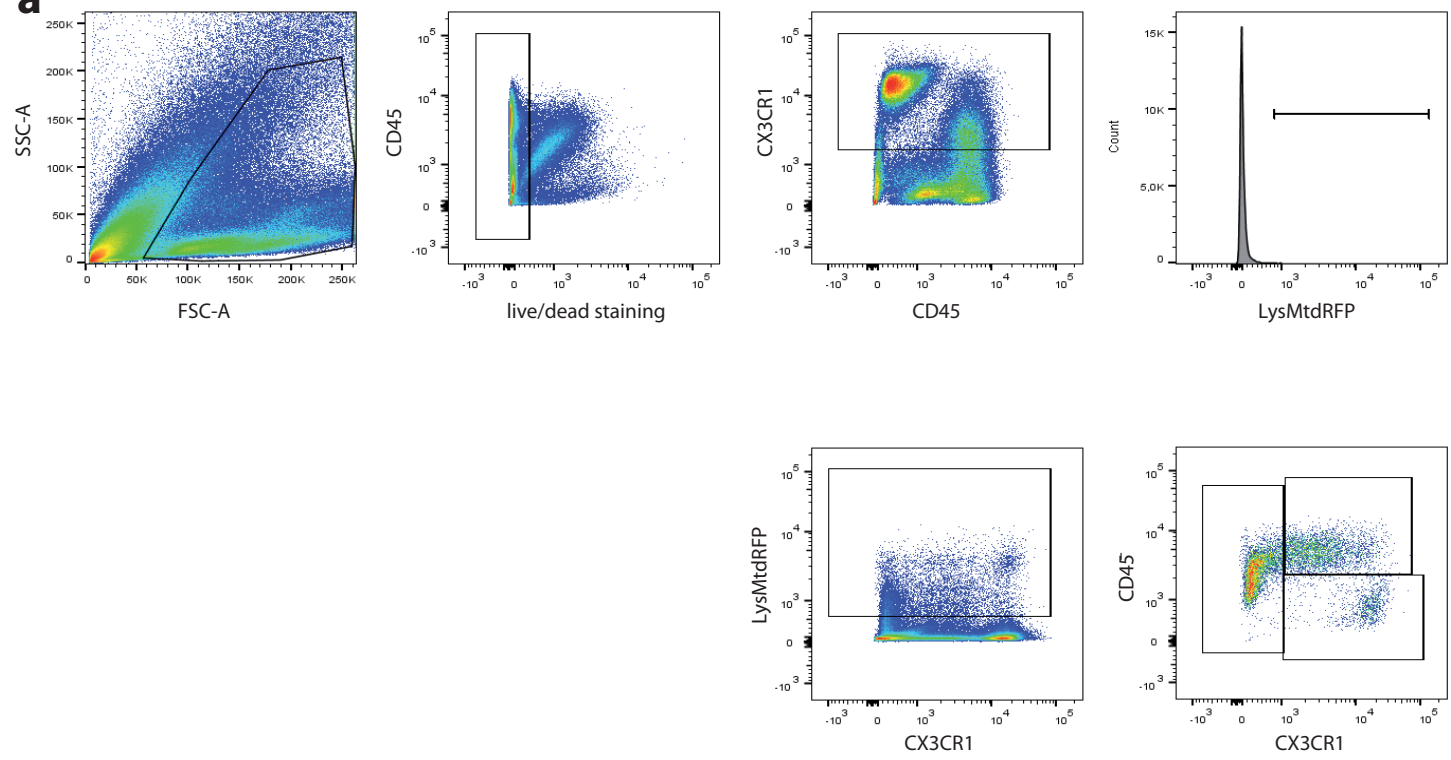

**b**

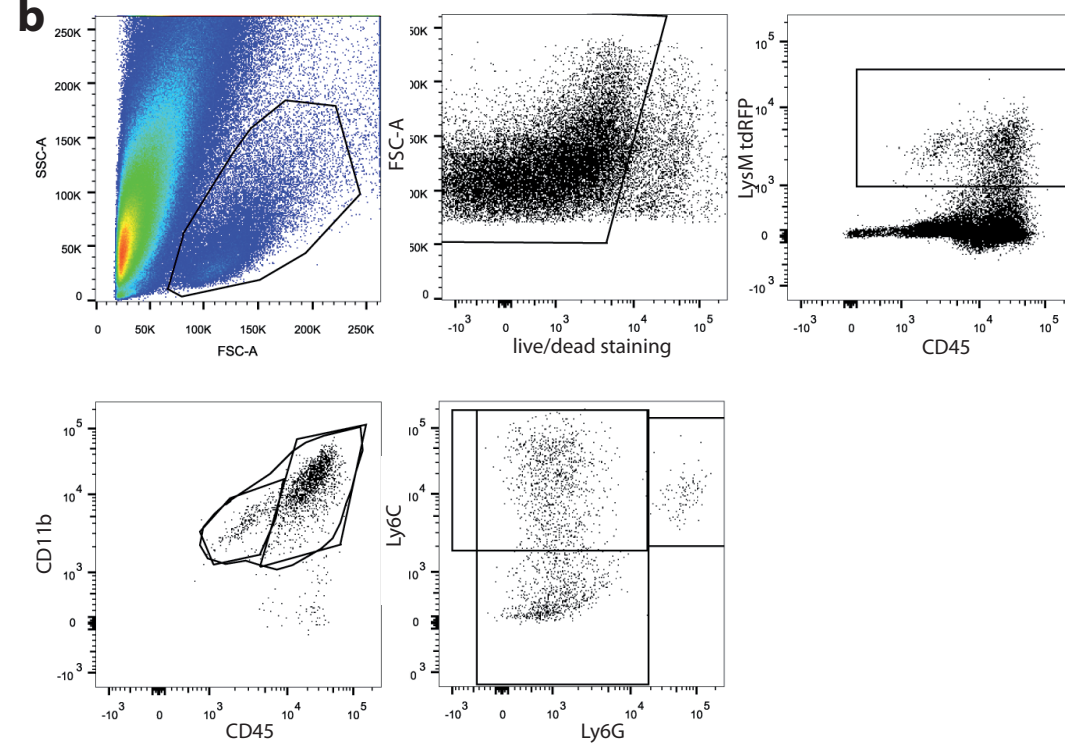

Supplement: Supplementary file 8 — Supplementary material 8 (PDF 685 kb) [file 401_2015_1497_MOESM8_ESM.pdf]
